# The cryo-EM structure of the endocytic receptor DEC-205

**DOI:** 10.1074/jbc.RA120.016451

**Published:** 2020-12-03

**Authors:** Benjamin S. Gully, Hariprasad Venugopal, Alex J. Fulcher, Zhihui Fu, Jessica Li, Felix A. Deuss, Carmen Llerena, William R. Heath, Mireille H. Lahoud, Irina Caminschi, Jamie Rossjohn, Richard Berry

**Affiliations:** 1 Infection and Immunity Program, Department of Biochemistry and Molecular Biology, Biomedicine Discovery Institute, Monash University, Clayton, Victoria, Australia; 2 Australian Research Council Centre of Excellence for Advanced Molecular Imaging, Monash University, Clayton, Victoria, Australia; 3 Ramaciotti Centre for Cryo Electron Microscopy, Monash University, Melbourne, Victoria, Australia; 4 Monash Micro Imaging, Monash University, Clayton, Victoria, Australia; 5 Department of Microbiology and Immunology, The Peter Doherty Institute, University of Melbourne, Parkville, Victoria, Australia; 6 Australian Research Council Centre of Excellence for Advanced Molecular Imaging, University of Melbourne, Parkville, Victoria, Australia; 7 Institute of Infection and Immunity, Cardiff University School of Medicine, Heath Park, Cardiff, United Kingdom

**Keywords:** immunology, cryo-electron microscopy, receptor structure function, oligomerization, membrane protein, receptor endocytosis, dendritic cell, CpG, cytosine–guanosine, CTLDs, C-type lectin-like domains, CysR, cysteine-rich, DCs, dendritic cells, ECDs, ectodomains, eGFP, enhanced green fluorescent protein, FNII, fibronectin type II, MR, mannose receptor, PLA2R, M-type phospholipase A2 receptor, SEC-MALS, size exclusion–coupled multiangle light scattering, SV-AUC, sedimentation velocity analytical ultracentrifugation

## Abstract

DEC-205 (CD205), a member of the macrophage mannose receptor protein family, is the prototypic endocytic receptor of dendritic cells, whose ligands include phosphorothioated cytosine–guanosine oligonucleotides, a motif often seen in bacterial or viral DNA. However, despite growing biological and clinical significance, little is known about the structural arrangement of this receptor or any of its family members. Here, we describe the 3.2 Å cryo-EM structure of human DEC-205, thereby illuminating the structure of the mannose receptor protein family. The DEC-205 monomer forms a compact structure comprising two intercalated rings of C-type lectin-like domains, where the N-terminal cysteine-rich and fibronectin domains reside at the central intersection. We establish a pH-dependent oligomerization pathway forming tetrameric DEC-205 using solution-based techniques and ultimately solved the 4.9 Å cryo-EM structure of the DEC-205 tetramer to identify the unfurling of the second lectin ring which enables tetramer formation. Furthermore, we suggest the relevance of this oligomerization pathway within a cellular setting, whereby cytosine–guanosine binding appeared to disrupt this cell-surface oligomer. Accordingly, we provide insight into the structure and oligomeric assembly of the DEC-205 receptor.

The macrophage mannose receptor (MR) protein family constitutes a group of large (∼160–200 kDa) membrane-bound multidomain receptors that includes the MR (1), the M-type phospholipase A_2_ receptor (PLA_2_R) (2), Endo180 (3), and DEC-205 (4). The MR protein family is characterized by a conserved domain architecture that comprises an N-terminal cysteine-rich (CysR) domain, a fibronectin type II (FNII) domain, and 8 or 10 contiguous C-type lectin-like domains (CTLDs). In addition, each receptor possesses consensus motifs within their cytoplasmic regions that facilitate their recruitment to clathrin-coated pits and ultimately allow continuous endocytosis of exogenous cargo to the cell interior (5). Although structurally related, the MR family exhibits a broad range of functions spanning phagocytosis of pathogens, internalization of soluble enzymes (PLA_2_R) or antigens (DEC-205).

DEC-205 (LY75 or CD205) is predominantly found on dendritic cells (DCs) (4, 6), specialized phagocytes that bridge the adaptive and innate immune compartments and function to sample exogenous ligands (7). Here, DEC-205 is thought to function as a promiscuous endocytic receptor that captures a diverse repertoire of foreign and self-antigens and internalize them for antigen processing and cross-presentation (8). Unlike other MR family members, DEC-205 is not predicted to bind sugars, as it lacks key residues implicated in oligosaccharide binding (9) but does mediate the internalization of phosphorothioated cytosine–guanosine (CpG) oligonucleotides (10), a widely used vaccine adjuvant (11). However, CpG oligonucleotides may not represent the natural ligand of DEC-205 because of their phosphorothioation at every position of the nucleotide backbone. In addition, DEC-205^+^ DCs are involved in the sensing of necrotic and apoptotic cells in a manner thought to promote peripheral tolerance (12). Indeed, DEC-205 is implicated in the detection of cell-death in tumor settings (13) with DEC-205 suggested to play a pH-dependent role in the binding of apoptotic cells *via* the recognition of the C-terminal tails of Keratin 1 and 10 upon cell death (14), Despite the diverse functional roles of the MR family, they seem to be united in their regulation by pH-induced structural rearrangements. Namely, DEC-205, PLA_2_R, and the avian IgY receptor FcRY are all reported to exist in a compact conformation at low pH and transition to an extended state under basic conditions (13, 15, 16), although the mechanism of such rearrangements are unknown. Further, the MR is thought to exist as a monomer in acidic conditions but multimerize at neutral pH (17). Indeed, such structural rearrangements are thought to influence receptor function with DEC-205 binding of keratins occurring in a pH-dependent fashion (14, 15). To understand the biological role of the MR family, high-resolution structural insight into such remodeling is required. While characterization of truncated MR family proteins have offered insight into ligand binding (18, 19), a complete picture of the receptor structure has remained unclear. Indeed, tracts of CTLDs, a conserved feature of the MR family, have been suggested to be too dynamic for high-resolution analysis. Further, cryo-EM studies of the MR family have been limited to low-resolution reconstructions (MR, 30 Å; Endo180, 25 Å; FcRY, 23 Å; DEC-205, 14.6 Å; PLA_2_R, 9.6 Å) (13, 15, 16, 17), which are insufficient to accurately place domains and highlights the dynamic nature of the MR protein family ectodomains (ECDs).

Here, we present the cryo-EM structure of the human DEC-205 monomer at 3.2 Å resolution, revealing a compact lemniscate structure formed of two concatenated rings of CTLDs. Further, we describe a pH-dependent and ligand-dependent oligomerization of DEC-205 *in vitro* as well as the cryo-EM structure of the DEC-205 tetramer at 4.9 Å resolution. The tetrameric form of DEC-205 showed an extensive remodeling of the secondary CTLD ring and suggested a role for the β2-3 loop of CTLD5 in mediating this transition state. The potential physiological relevance of the DEC-205 tetramer was suggested using molecular imaging techniques in the absence and presence of CpG ligand. Our structural analysis of DEC-205 provides a fundamental advance in our understanding of this promiscuous endocytic receptor and the mannose-receptor family more broadly.

## Results

### The cryo-EM structure of the human DEC-205

We produced and purified the soluble DEC-205 ECD (residues 1–1664 spanning the N-terminal CysR to the C-terminal CTLD10) from mammalian expression to yield protein that was well behaved and suitable for structural studies. Given that DEC-205 adopts a compact conformation at low pH (13), we prepared samples for single-particle cryo-EM data collection at pH 6.0 consistent with early late endosomes where DEC-205 traffics.

**Figure 1:**
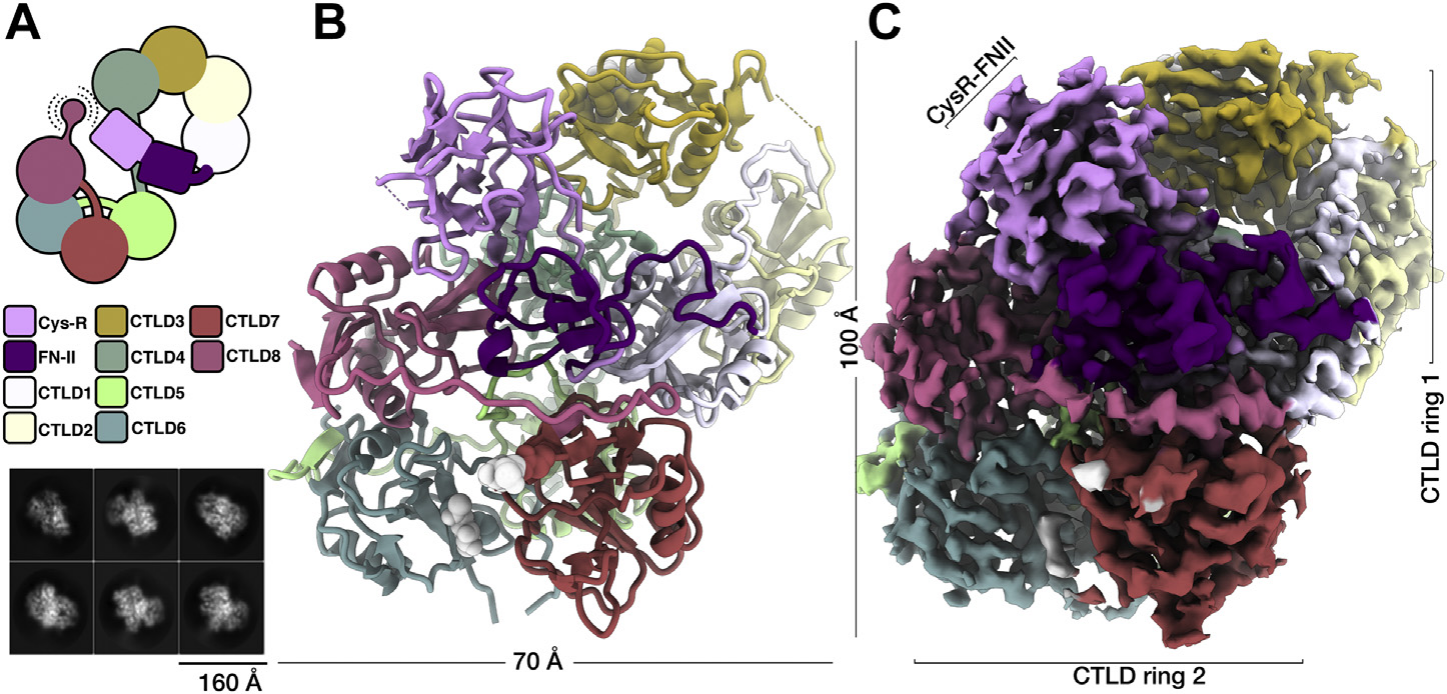
**Overview of the DEC-205 cryo-EM monomer structure.***A*, schematic representation of the DEC-205 structure showing the domain layout with the proboscis showing the mobile C-terminus and corresponding two-dimensional cryo-EM class averages of the DEC-205 monomer. *B*, the cryo-EM reconstruction of the DEC-205 monomer colored by domain subunits shown in cartoon and the cryo-EM reconstruction. *C*, the DEC-205 monomer formed a lemniscate structure formed by intercalating rings of C-type lectin domains (CTLDs) with the cysteine-rich (Cys-R) and fibronectin (FN) domains sat atop the intersect. Domains colored Cys-R (*pink*), FN (*purple*), CTLD1-8 (*white*, *light yellow*, *gold*, *aqua*, *light green*, *dark green*, *salmon*, and *red*, respectively).

The structure of the DEC-205 ECD was determined to a resolution of 3.2 Å (Fig. 1, Table 1 and Fig. S1). The cryo-EM reconstruction allowed unambiguous placement of the individual DEC-205 domains with almost contiguous density throughout the reconstruction. The DEC-205 ECD domains pack tightly together presenting in a lemniscate (∞) with dimensions of roughly 110 × 70 × 70 Å. Each of the tightly packed DEC-205 ECDs could be built from the N-terminal CysR (R30) to CTLD8 (E1383); however, the C-terminal CTLD 9 and 10 were invisible in the reconstruction. A large proportion of sidechains evident throughout the 2.5 Å core of the protein (Fig. 1 and Fig. S2). While the surface of the reconstruction was slightly lower resolution (∼3.8 Å), it was still sufficient to allow visualization of the extended interdomain linkers (Fig. S2*A*) and N-linked glycans (N529, 1076, and 1158, and an apparent glycosylation at an N-X-C site at 1253)(Fig. S2*B*).

**Table 1:** Cryo-EM data collection, refinement, and validation statistics

Data collection, refinement, and validation parameters	DEC-205 monomer (EMDB 22422, PDB 7JPT)	DEC-205 tetramer (EMDB 22423, PDB 7JPU)
Data collection and processing		
Magnification	130,000	130,000
Voltage (kV)	300	300
Electron (e^−^/Å^2^)	53	63
Defocus range (μm)	−0.8 to −2.8	−0.8 to −3.5
Pixel size (Å)	0.53	1.06
Symmetry imposed	C1	D2
Initial particle images (No.)	610,069	185,187
Final particle images (No.)	310,803	102,569
Map resolution (Å)	3.2	4.9
FSC threshold	0.143	0.143
Map resolution range (Å)	2.9–3.7	4.2–7.0
Refinement		
Initial models (PDB ID)	CysR (1DQO) (19), FN (2V5P) (40), and CTLD (1QDD) (41)	CysR (1DQO) (19), FN (2V5P) (40), and CTLD (1QDD) (41)
FSC threshold	0.143	0.143
Map sharpening B factor (Å2)	−140	−290
Model composition and validation		
Nonhydrogen atoms	10,220	35,992
Protein residues	1327	4631
Ligand	884.84	-
B factors (Å)		
Protein	83.43	147.81
Ligand	104.87	-
R.m.s deviations		
Bond lengths (Å)	0.002	0.002
Bond angles (°)	0.524	0.583
Validation		
Molprobility score	2.31	1.97
Clashscore	8.51	9.85
Poor rotamers	3.48	0.00
Ramachandran		
Favored (%)	93.16	92.92
Allowed (%)	6.77	7.08
Disallowed (%)	0.08	0.00

FSC, fourier shell correlation.

**Figure 2:**
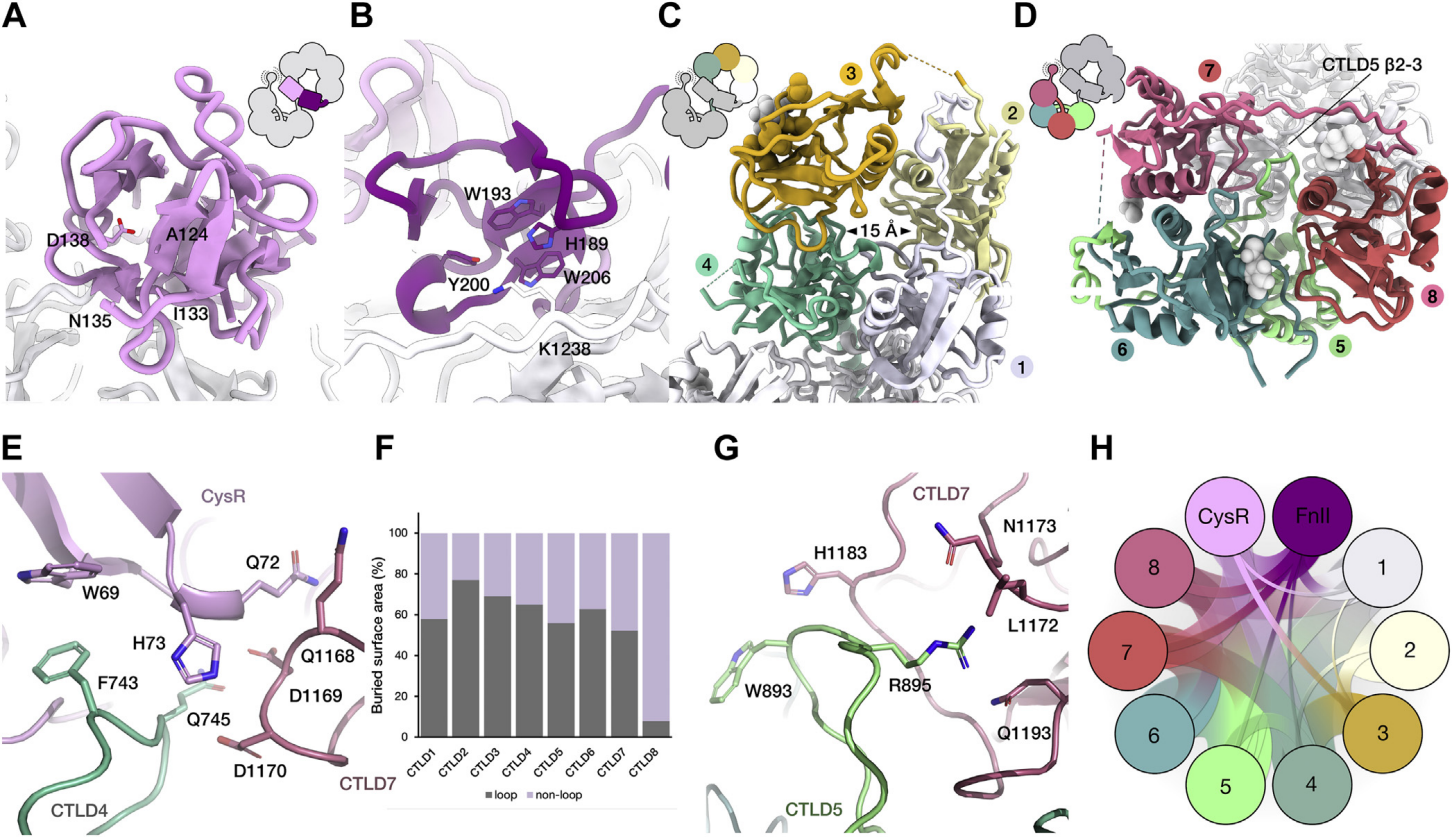
**Structural characterization of the DEC-205 monomer.** Overview of the DEC-205 monomer structure. *A*, an enlarged view of the Cys-R (*pink*) and FN, (*B*) (*purple*) domains sat atop the central intersect. An enlarged view of lemniscate ring 1 comprising CTLD domains 1 to 4 (shown in *white*, *light yellow*, *gold*, and *aqua*, respectively), shown (*C*). An enlarged ring 2 comprising CTLD domains 5 to 8 (shown in *light green*, *dark green*, *salmon*, and *red*, respectively), shown (*D*). *E*, enlargement of the CysR (*pink*) interaction with CTLD4 (*aqua*) and CTLD7 (*salmon*). *F*, breakdown of the buried surface area contributions from the CTLDs and highlighting the large contribution from the β2-3 loops. *G*, enlargement of the extended and charged β2-3 of CTLD5 (*light green*) interactions with CTLD7 *(salmon*). *H*, buried surface area (BSA) analysis of the DEC-205 monomer. Representation of the interdomain DEC-205 interactions within the monomer where the width of the line corresponds to the BSA in Å^2^ as derived from the PDBePISA software. CTLD, C-type lectin domain; Cys-R, cysteine-rich; FN, fibronectin.

The overall architecture of the lemniscate comprised of two interwoven rings formed by CTLD1-4 and CTLD5-8, with the CysR and FNII domains located above the central intersect (Fig. 2, *A*–*B*). The centrally located and globular CysR and FNII domains lead into a compact 60 Å ring with a central cavity of ∼15 Å comprised of a counter-clockwise arrangement of CTLD1 through to CTLD4 (Fig. 2*C*). The second and slightly larger ∼65 Å ring comprises of a clockwise arrangement of CTLD5 through to CTLD8. Here, the central cavity of the second ring was occupied by an extended loop of CTLD5 (Fig. 2*D*). Although the reconstruction was of high quality, surprisingly CTLD9 and CTLD10 were lacking in the final reconstruction, although Western blot targeting the C-terminus confirmed intact protein, and no signs of degradation post imaging (data not shown). Inspection of the 2D class averages (Fig. 1) indeed shows an element of flexibility at the C-terminus; thus, we conclude it is highly mobile. Further, the recent structure of a truncated form of the MR could not determine the position of the C-terminal CTLD because of it being mobile in the crystal structure (20). Taken together, these data suggest that C-terminal CTLDs can be structurally dynamic and may allow the ECD freedom to sample for ligands or antigens.

Comprehensive comparisons to the previously described low-resolution DEC-205 model was not possible given a lack of model coordinates; however, the model did identify the double-ringed architecture, although 8 out of 12 domains were incorrectly placed (Fig. S3) (13).

### Structural insights into the DEC-205 domains

The CysR domain of DEC-205 adopted the classical β-trefoil architecture of the domain family (Fig. 2*A*), comprising of 12 antiparallel β-sheets with a loose two-fold internal symmetry (Fig. S4*A*). Each of the tripartite nodes includes four short β-sheets β1-4, β5-8, and β9-12, respectively, that are stabilized by three anchoring disulfide bonds akin to the CysR domain of the MR (21). A notable observation for the MR CysR was the identification of a pocket formed by the β11 to 12 loop and neighboring residues (19, 21). This pocket was shown to bind the sulfate group of sulphated carbohydrates, a proposed ligand of the MR (19). A similar pocket is found within the DEC-205 CysR domain, and the location of the domain at the intersect of the two CTLD rings could allow ligand to the pocket comprised of A124, I133, N135, and D138 (Fig. 2*A*). However, although the β11 to 12 loop of the DEC-205 CysR formed a similar confirmation to that in the MR, the deletion of the ligand coordinating N102 found in the MR would likely render DEC-205 unable to coordinate sulfated ligands, corroborating previous findings (19).

The FNII of DEC-205 formed a compact domain formed by two antiparallel β-sheet stacks of β1-2 and β3-4 which are tethered by two disulfide bonds that bridge over β3-4 to stabilize neighboring loops (Fig. 2*B*). Overall, the domain closely resembled that of the recently solved MR FNII notwithstanding subtle movements within the connecting loops (Fig. S4*B*) (20). A hydrophobic groove first observed in the MR FNII and subsequently in the related Endo180 FNII (22) has been implicated in conferring collagen binding. In DEC-205, the hydrophobic groove along the surface of the domain formed by H189, W193, Y200, and W206 persists. However in the context of the DEC-205 ECD, the hydrophobic pocket is partially occluded by the linker between CTLD 7 and 8, meaning key residues thought to dictate collagen reactivity in the MR would not be available for ligand binding (Fig. 2*B*) (20).

A defining feature of the MR family is the tracts of CTLD domains, here each of the DEC-205 CTLDs adopted the prototypic double loop structure of the CTLD fold (23), comprising of two α-helices encasing an antiparallel stack of five β-sheets that was conserved throughout with a root mean squared deviation of ∼1.7 ± 0.7 Å, (Fig. S4, *C*–*J*). Each of the CTLDs adopted a compact β2-3 loop architecture with the exclusion of CTLD5 that stood in stark contrast with a fully extended β2-3 loop that played a role in coordinating the secondary CTLD ring (Fig. S4*G*) and thus in stabilizing the DEC-205 ECD. The first CTLD ring of the DEC-205 ECD had a 15 Å cavity with no loop plugging the core to serve a similar coordinating role. Owing to divergent amino acid sequences, the 10 contiguous CTLDs of DEC-205 are thought to be a structural scaffolds for ligand binding and are not predicted to bind calcium ions which facilitate oligosaccharide binding in other CTLDs (Fig. S5) (9). Indeed, the β2-3 loops and the β3 of each respective CTLD domain lack the necessary sidechain chemistry needed to coordinate calcium ions which coordinate vicinal hydrogen bonds that impart sugar-binding capacity to other CTLDs (Fig. S5). Notably, the reconstructed map showed no unambiguous evidence of coordinated sugars or Ca^2+^ although we cannot exclude this possibility.

**Figure 3:**
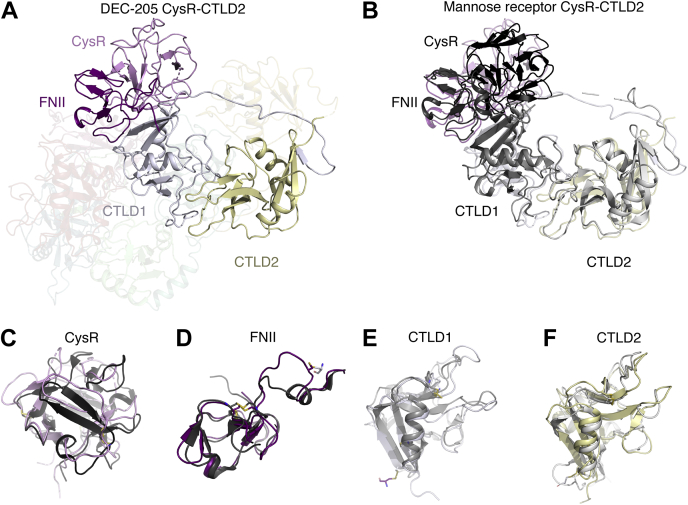
**Comparison of the DEC-205 CysR to CTLD2 with the mannose receptor.***A*, structural comparison of the DEC-205 cysteine-rich domain (CysR), fibronectin type II (FNII), C-type lectin-like domain (CTLD) 1 and 2 and alignment to the respective domains within the macrophage mannose receptor (PDB-5XTS) (*B*). Enlargements of the cysteine-rich domain, fibronectin type II, and C-type lectin-like domain 1 and 2 domain alignments (*C*–*F*).

Structural comparison to the structure of a truncated N-terminal fragment of the MR showed a high amount of structural conservation with ∼2.2 Å root mean squared deviation when aligning to the corresponding CysR-CTLD2 of the DEC-205. Within the respective structures, there are subtle domain movements, most notably in the CysR that rotates 8° and moves 2 Å in the context of the DEC-205 ECD relative to the truncated MR structure (Fig. 3, *A*–*B*). Similarly, the FNII domain moves 9° and translates 5 Å in the DEC-205 ECD, whereas the CTLD1 and 2 rotate ∼6° and move 5 and 2 Å, respectively, although the individual domains are relatively similar (Fig. 3, *C*–*F*).

In short, the cryo-EM structure of the DEC-205 ECD is of high quality, revealing the atomic architecture of the mannose-receptor family in new light and will serve as a platform for further characterization of DEC-205

### Interdomain interactions within the DEC-205 ECD

The compact DEC-205 ECD was stabilized by an extensive network of interdomain interactions. Given the central position of the CysR domain, it made numerous contacts to CTLD4 and 7 and the neighboring FNII. The contacts from the CysR includes Q72 and H73 of the β4-5 loop that projects toward the CTLD rings and are buried within Q1168-D1170 of the retracted β2-3 loop of CTLD7 (Fig. 2*E*). Further, W69 of the β4 sheet of the CysR accommodates F743 of CTLD4, which projects up from the first CTLD ring (Fig. 2*E*). The neighboring Q745 of CTLD4 contacts Q1168-D1170 of the retracted β2-3 loop of CTLD7 to stabilize the interface around H73 (Fig. 2*E*). Although a much more compact domain, the FNII made peripheral contact to CTLD7 and 8 but mainly interacts with the α1 and face of the β1, driven by a salt bridge from R167 (FNII) to E242 (CTLD1). In addition to the extensive contacts from the CysR and the FNII, CTLD1 made extensive contact to the terminal CTLDs of the two rings, namely CTLD4 and 8, which serves to stabilize the first CTLD ring. Instead of calcium-binding, the CTLD β2-3 loops seem to have been repurposed toward stabilizing the overall arrangement of the DEC-205 ECD. In fact, around ∼50% of the total interdomain buried surface area (BSA) of the DEC-205 ECD is accounted for by β2-3 loop-mediated interactions (Fig. 2*F*). Most striking was the extended β2-3 loop of CTLD5, which acted as a “recruitment hub”, whereby it co-ordinated several domains within the secondary ring including CTLDs 6, 7, and 8. Intriguingly, the β2-3 loop of CTLD5 contained an apical charged cluster of, W893, H894, and R895 that interacted with CTLD7. Here, R895 seemed to tether the loop apex *via* sidechain interaction with a neutral pocket composed of L1172, N1173, and Q1193 within CTLD7 with peripheral interaction between W893 and H1183, which further stabilized the extended long-loop conformation (Fig. 2*G*).

The extensive interdomain BSA and interactions of the DEC-205 ECD included major contributions from the central domains of the lemniscate, namely CysR, CTLD1, CTLD4, and CTLD5 and 8 (summarized Fig. 2*H* and Table S1). The cryo-EM structure of the DEC-205 ECD provided profound insight into the structural arrangement of a MR family member. Moreover, the high-resolution of the DEC-205 ECD allowed unambiguous placements of the domains and robust placement of the sidechains beyond those previously identified for DEC-205 (13), Endo180 (17), and the human M-type phospholipase A2 receptor (16).

### DEC-205 undergoes pH-responsive oligomerization

While optimizing DEC-205 for high-resolution cryo-EM studies, we identified the propensity for DEC-205 to oligomerize in a pH-dependent manner. As seen with other members of the MR family, such as the MR, DEC-205 can be structurally dynamic and pH labile (20). Oligomerization has also been observed for more distantly related members of the C-type lectin-like proteins including NKR-P1, which forms dimers and higher-order oligomers both *in vitro* (24, 25) and *in vivo* (26). Furthermore, the human MR can undergo pH-dependent conformational changes that impact collagen binding, corroborating earlier observations of pH-dependent conformational changes in the Endo180 receptor (17). As such, we set out to identify the molecular basis for the oligomerization of DEC-205.

**Figure 4:**
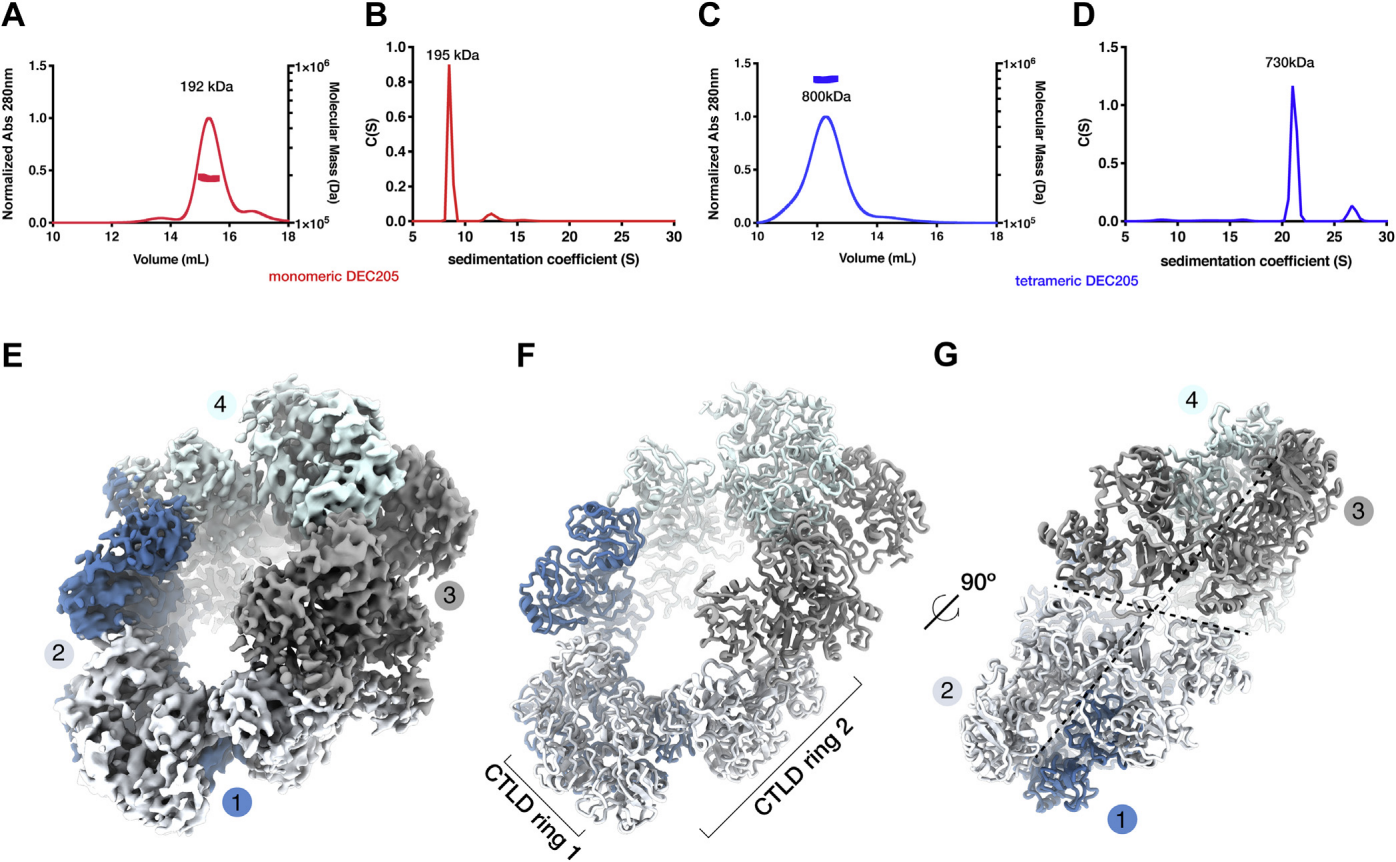
**Biophysical characterization of the DEC-205 pH-dependent oligomerization and structure of the DEC-205 homotetramer.***A*, size exclusion–coupled multiangle light scattering (SEC-MALS) chromatograph of DEC-205 at pH 6.0 shows DEC-205 monomers, corroborated by sedimentation velocity analytical ultracentrifugation (SV-AUC) shown (*B*). Biophysical characterization of DEC-205 at pH 8.0 identified a tetramer as shown by SEC-MALS (*C*), and corroborated by SV-AUC (*D*). Overall architecture of the DEC-205 tetramer. *E*, overall view of the DEC-205 tetramer cryo-EM reconstruction with the four DEC-205 protomers colored *blue*, *light blue*, *cyan*, and *gray*. *F*, the cartoon depiction clearly shows the toroid structure with ring 1 of DEC-205 forming a head–head interaction at the extreme poles of the toroid with the tail–tail interaction forming across the equator. *G*, the clear D2 symmetry of the interaction became clear during classification and refinement of the reconstruction.

Utilizing size-exclusion chromatography coupled to multiangle light scattering, purified DEC-205 ECD at acidic conditions (pH 6.0) reproducibly formed a monomer with a mass in agreement with the predicted mass (192 kDa as derived from amino acid sequence) (Fig. 4*A*). Notably, these biophysical measurements further validated that the protein being produced was intact and simply mobile within the cryo-EM reconstruction. Next, we employed sedimentation velocity analytical ultracentrifugation (SV-AUC) to further investigate the oligomeric state of the DEC-205 ECD at acidic pH. Similarly, DEC-205 existed as a monomer at pH 6.0 (S_20,w_ = 8.6, *f/f*_*o*_ = 1.36) with a small amount of dimer evident (Fig. 4*B*). Building on this finding, we observed a large change in the elution profile of DEC-205 at pH 8.0, eluting with a mass four times that of the monomer, suggesting that in basic conditions DEC-205 formed a stable tetramer (Fig. 4*C*). Further SV-AUC experiments confirmed the presence of a stable 730 kDa DEC-205 tetramer (S_20,w_ 21.7 (*f/f*_*o*_ 1.4),) with a small proportion of 1020 kDa hexamer (S_20,w_ 27.6, *f/f*_*o*_ 1.5) (Fig. 4*D*). Further, we investigated the monomeric-oligomeric state of DEC-205 at neutral pH and found that the vast majority was monomeric with a consistent yet reproducible component of tetramer. Together, these data show that DEC-205 can oligomerize *in vitro* from a stable monomeric form into a tetramer in response to a change in environmental pH.

### DEC-205 forms a homotetrameric structure

We next performed cryo-EM analysis on the DEC-205 tetramer in basic conditions (pH 8.0). Here, the DEC-205 tetramer formed a 115 × 200 × 100 Å toroid shaped particle with a large central cavity (Fig. 4, *E*–*G*) and was ultimately resolved to 4.9 Å (Fig. S6).

**Figure 5:**
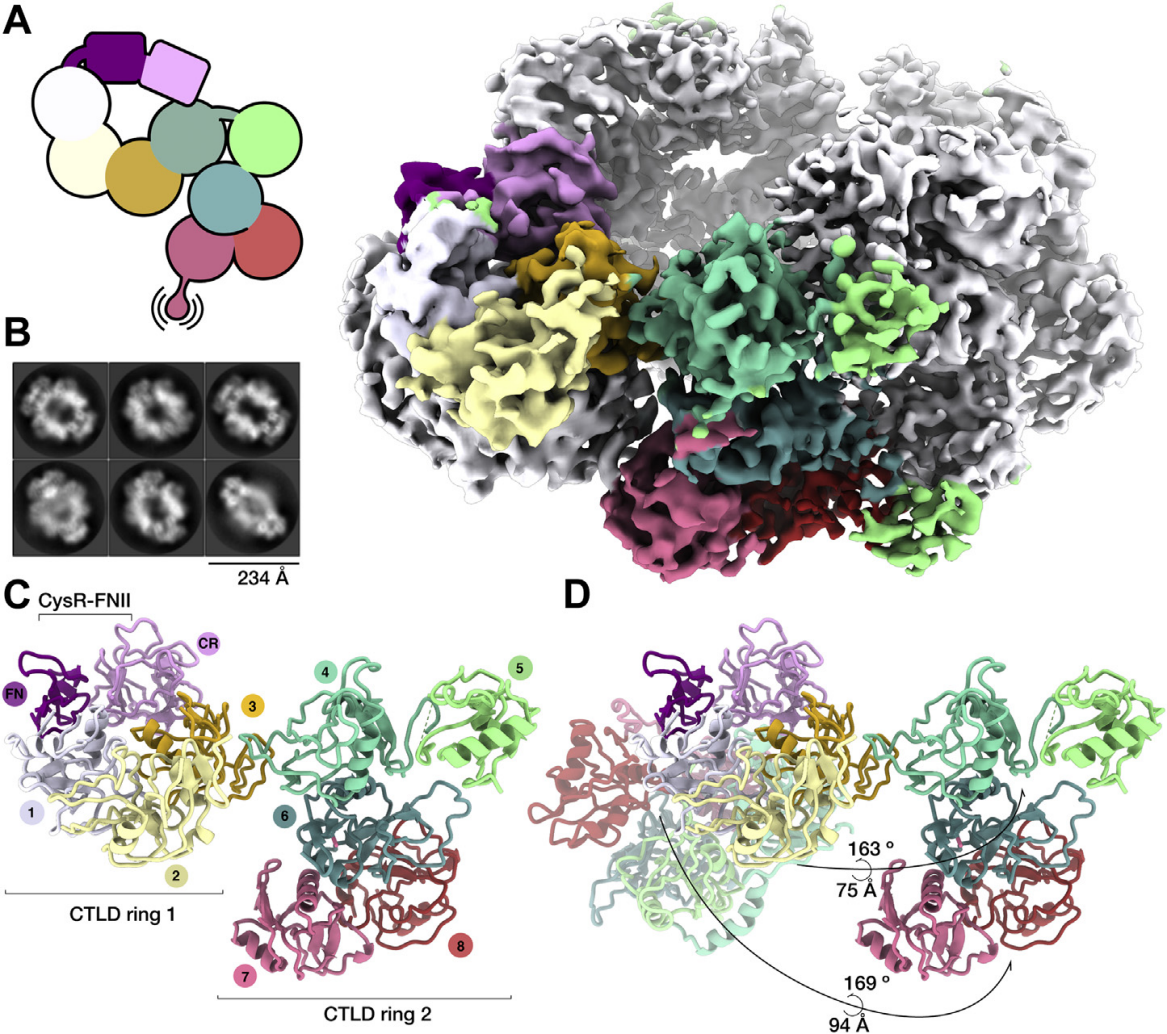
**DEC-205 undergoes significant restructuring to form a homotetramer.***A*, the cryo-EM reconstruction of the DEC-205 tetramer colored by chain for the four protomers with one chain colored by domain as in Figure 1 and a schematic overview of the DEC-205 protomer within the tetramer structure. *B*, two-dimensional cryo-EM class averages of the DEC-205 tetramer. *C*, an enlargement of a single DEC-205 protomer from the tetramer shows the unfurling of the compact lemniscate structure to form the head to head – tail to tail interactions that stabilize the tetramer. *D*, the structural rearrangement required for oligomerization involves a dramatic movement of the CTLD ring 2, between ∼75 and 95 Å for the CTLD domains 5 to 8 and a rotation of ∼165°, shown overlaid with the monomeric DEC-205 structure. CTLD, C-type lectin-like domain.

The overall architecture of the DEC-205 tetramer revealed a major rearrangement of the second CTLD ring (Fig. 5*A*) that enabled four protomers to assemble into a prolate toroid with clear D2 symmetry in the 2D class averages and final reconstruction (Fig. 5*B*). Here, the Cys-R, FNII, and CTLD1-3 remained globally unchanged within the first CTLD ring and stacked against the corresponding ring of another protomer such that the CysR and FNII domains of the respective protomers were exposed (Fig. 5*C*). Notably in this confirmation, the hydrophobic groove within the FNII domain was no longer occluded by the CTLD7-8 linker.

In contrast to the structural conservation of the first ring, the C-terminus underwent a dramatic rearrangement to form a tail–tail interaction along the median of the toroid. Here, the second CTLD ring comprised of CTLD5-8, unfurled 130 Å away from ring 1 to form a much more extended arrangement (Fig. 5*D*). Within this extended confirmation, CTLD8 from one protomer contacted CTLD4 of the opposing protomer and vice versa. The cryo-EM reconstruction was unambiguous for the placement of the 10 (CysR to CTLD8) domains except for CTLD5. Here, the cryo-EM reconstruction suggests that CTLD5 is highly mobile within the tetramer, and the domain could act as an allosteric center for DEC-205’s pH-dependent rearrangements. Following localized refinement of the reconstruction, it enabled overall domain placement of CTLD5, but it was not possible to determine the conformation of the β2-3 loop (Fig. S6*B*). As observed in the DEC-205 monomer, CTLD9 and 10 were absent from the final reconstruction, solidifying the notion that they are a highly dynamic part of the protein. The BSA for the tetramer was much more extensive, and the modest resolution did not allow atomic analysis of the interfaces. Broadly the BSA of the protomer 1 and 2 head–head interaction accounted for ∼2300 Å^2^, whereas the protomer 1 and 3 tail—tail interaction was ∼1500 Å (Fig. S7 and Table S2). Mainly these interactions centered around CysR-CTLD3 protomer interactions for the head–head and CTLD4-8 for the tail–tail. The cryo-EM structure of the DEC-205 tetramer illustrates the extensive structural rearrangements undertaken in the monomer–tetramer transition and provides the higher-order arrangements of this protein family in new clarity.

### DEC-205 cell surface oligomeric state and CpG binding

Our results indicated that the soluble DEC-205 ECD formed a pH-dependent structure *in vitro*, but it remained unclear how DEC-205 resided on a cell surface. Although an endocytic receptor with a known role in endosomal recycling (8), we next set out to investigate the cell surface context for the DEC-205 receptor using confocal microscopy.

**Figure 6:**
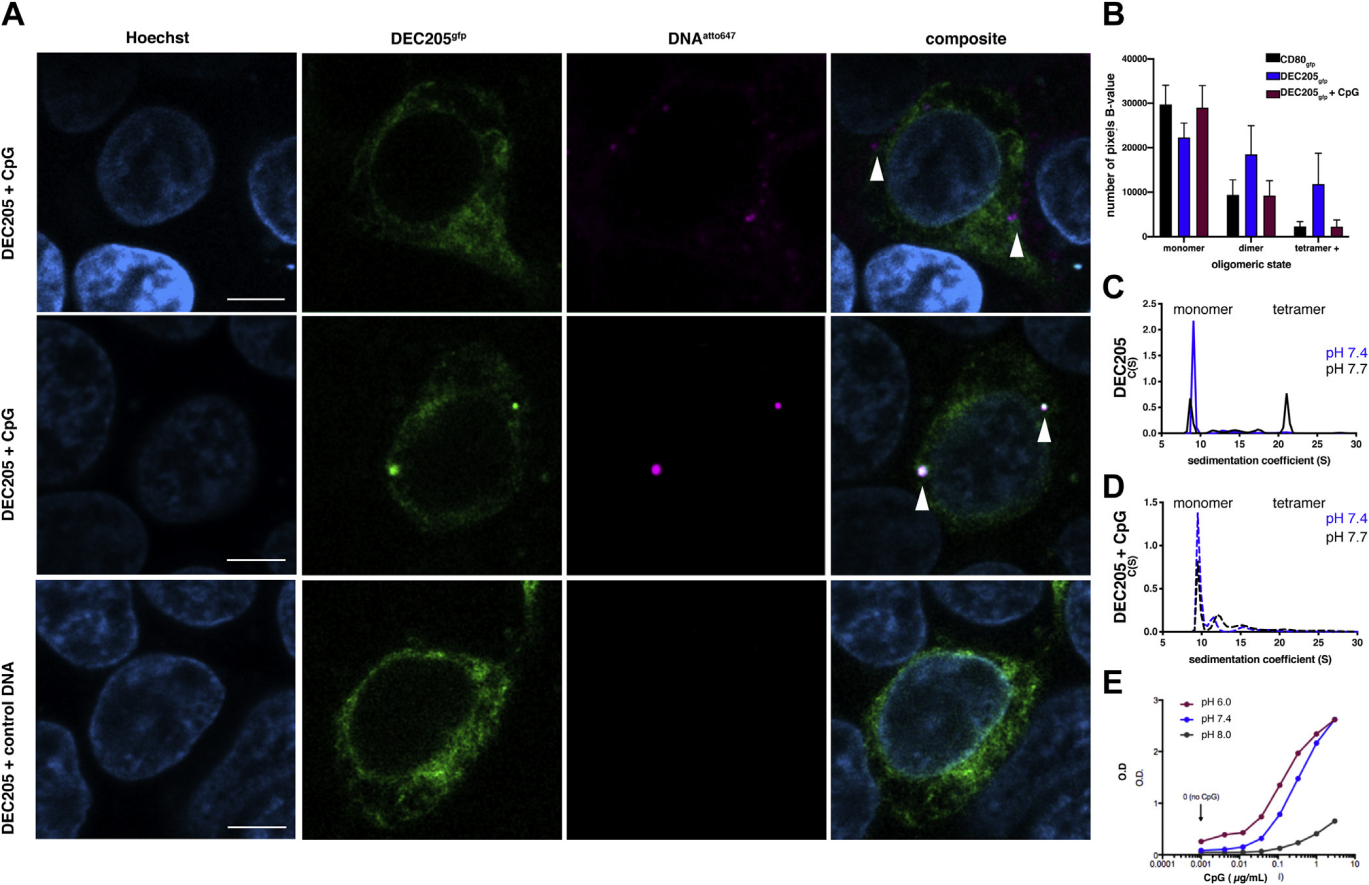
**Biological validation of the DEC-205 tetramer and impact of CpG binding upon monomerization.***A*, representative confocal images of DEC-205_gfp_^+^ colocalization with CpG (*magenta*, atto647) both formed puncta (*middle row*) within the cell and more diffuse staining on the cell surface (*right row*) with no interaction present with a CpG control (*left row*). CpG binding by DEC-205 is oligomerization state specific. Scale bar = 5 μM. *B*, number and Brightness (N&B) microscopy analysis of CD80_gfp_^+^ and DEC-205_gfp_^+^ cells. Although both CD80_gfp_^+^ and DEC-205_gfp_^+^ HEK293T cells exhibit similar average intensity patterns, DEC-205 clearly exists in larger oligomeric assemblies on a cell surface. N&B number of pixel B-values shows the overall trend analysis of CD80_gfp_, and DEC-205_gfp_ shows a shift from equal abundance of DEC-205 monomer, dimer, and oligomers on a cell surface shifts to ∼73% monomer in the presence of CpG, similar to that of CD80_gfp_ (Values are the mean value for n = 7 replicates, and error bars denote SEM). *C*, SV-AUC data on DEC-205 show a fine pH threshold for monomer-tetramerization transition. Incubation with CpG shifts tetramerized DEC-205 to monomer (*D*). *E*, ELISA assays with immobilized DEC-205 confirm pH responsive binding to CpG. CpG, cytosine–guanosine; SV-AUC, sedimentation velocity analytical ultracentrifugation.

We first sought to establish whether cell surface DEC-205 could bind to the CpG oligonucleotide 1668 (5′TCC ATG ACG TTC CTG ATG CT), a previously established DEC-205 ligand (10). Cells expressing membrane-bound full-length DEC-205 (FL-DEC-205, residues 1–1722) in-frame with a C-terminal enhanced green fluorescent protein (eGFP) showed strong cell surface and endosomal staining. To monitor the ability of cell surface FL-DEC-205 to bind CpG, we undertook confocal microscopy in the presence of CpG with a 3’Atto647 fluorophore. Here, cell surface FL-DEC-205 showed strong binding to CpG and colocalized diffusely on the cell surface and more notably within subcellular endosomal compartments suggesting CpG internalization (Fig. 6*A*). Further, the specificity of interaction was shown to be specific for the phosphorothioate of the CpG ligand, whereby a nonthioated CpG control showed no cellular staining.

We next sought to determine the impact of DEC-205 oligomeric state on the cell surface using number and brightness (N&B) fluctuation spectroscopy analysis. N&B analysis is a method that measures both the mean and variance of fluorescence intensity distributions per pixel from a stack of images to deconvolute the number of molecules (N) and their brightness (B), where B directly relates to the oligomeric state (27). Conducting N&B analysis on cells expressing FL-DEC-205_gfp_, we directly quantified the oligomeric state of DEC-205 on a cell surface. Confocal scanning microscopy showed cell surface expression of FL-DEC-205 with B values consistent with the presence of monomers, dimers, and oligomers potentially corresponding to tetrameric DEC-205 (Fig. 6*B*). Higher-order species were also observed and are thought to correspond to a small observation of a hexameric form of FL-DEC-205 that was observed during the cryo-EM analysis of the DEC-205 tetramer (Fig. 6*B*). Notably, a control cell line expressing full length CD80_gfp_ exhibited similar average intensities; however, the predominating CD80 cell surface species was monomeric, in agreement with the well-documented monomeric nature of this receptor (Fig. 6*B*). Thus, our N&B analysis served to support the observation that FL-DEC-205 existed in both monomers and larger oligomers on a cell surface and provided context for our structural work.

To begin to address the notion of whether DEC-205 oligomerization had a functional role in ligand recognition, we next conducted N&B analysis in the presence of CpG oligonucleotide. Here, the addition of CpG induced a switch to mostly monomeric DEC-205 with a notable decrease in cell surface tetramers (Fig. S8). Indeed, overall trend analysis showed FL-DEC-205_gfp_^+^ cells shifted from an equal abundance of monomers, dimers, and oligomers (42, 35, and 22%, respectively) to a predominantly monomeric cell surface form (∼73%) in the presence of CpG (Fig. 6*B*). Thus, these microscopy data suggest that FL-DEC-205 resides on a cell surface in a state of structural equilibrium comprising of DEC-205 monomers and oligomers. Thus, these observations on the cell-surface DEC-205 support the structural work on the DEC-205 ECD. Furthermore, such observations provide a possible biological context for the monomer and tetrameric arrangements of DEC-205 identified in our biophysical and structural data.

Given how the oligomeric state of FL-DEC-205 was impacted by ligand binding investigated the impact of CpG oligonucleotide binding on DEC-205 oligomerization *in vitro*. SV-AUC analysis of the DEC-205 ECD at pH 7.4 showed a largely monomeric species with a minor component of higher-order species somewhat consistent with the cell surface FL-DEC-205(Fig. 6*C*). A subtle movement of the pH to 7.7 skewed the distribution toward an equal monomer–tetramer equilibrium; however, upon the addition of CpG, this shifted the DEC-205 ECD to a monomer (Fig. 6*D*). These data suggest that the monomeric form of DEC-205 is the ligand binding component. To decipher the impact of pH on the ligand-binding ability of DEC-205, we next conducted an ELISA assay at differing pH. Immobilized soluble DEC-205 ECD was then incubated with CpG at a pH 6.0, 7.4, and 8.0. DEC-205 binding to CpG was enhanced at acidic pH relative and neutral pH but diminished notably in alkaline conditions (Fig. 6*E*). Taken together, these data suggest that monomeric DEC-205 is the configuration that binds to CpG and that low pH is thus conducive to CpG binding because of disruption of the DEC205 oligomer.

## Discussion

The high-resolution structure of the DEC-205 ECD has provided insight into the atomic structure of the macrophage mannose-receptor family. In addition, characterization of the pH-dependent oligomerization of DEC-205 and the solving of the tetrameric DEC-205 structure showed a complex conformational remodeling occurs during oligomerization.

The DEC-205 monomer showed the lemniscate structure of DEC-205 ECD and macrophage MRs is comprised of two intercalated rings of CTLDs with the CysR and FN domain located at the central intersect. In addition, we discovered the ability of DEC-205 to form stable oligomeric species and solved the structure of the tetrameric form of DEC-205. The tetramer structure revealed a major restructuring of the second (C-terminal) CTLD ring that facilitated a head–head and tail–tail protomeric arrangement in the tetramer. Such structural rearrangement was directly resultant from an increase in pH, a trait observed in studies of the MR. Similar pH-dependent rearrangements were observed for another receptor containing multiple-repeat domains, *e.g.*, the insulin-like growth factor 2 receptor (28). Notably, the pH-dependent conformational plasticity was thought to enable ligand binding in one state on a cell surface and enable ligand dissociation upon further conformational changes following endosomal acidification (28). In particular, the pH-dependent nature of histidine sidechains was thought to orchestrate the observed conformational changes as we hypothesize histidines with the β2-3 loop of the CTLD5 “recruitment hub” may act. The cellular work conducted here supported the presence of DEC-205 tetramers on the surface of cells and established that CpG binding disrupted cell surface oligomers. Accordingly, the current data suggest that DEC-205 resides on a cell surface in a monomer–oligomer equilibrium, but it is the monomer that appears to be the “active” form for CpG engagement. Of interest, the formation of higher-order oligomeric forms of a C-type lectin has been observed before. The DC adhesin DC-SIGN is a C-type lectin previously observed to form cell-surface tetramers implicated in the high-avidity binding of the HIV envelope protein gp120 (29, 30). In addition, another study showed that the human MR exists as a dimer (31, 32, 33) with a follow-up study showing oligomers on the surface of macrophages and monocyte-derived DCs (34). Similar to DC-SIGN, MR binding to gp120 was enhanced *via* homo-oligomerization (34).

Given the structural conservation throughout the MR protein family, it is possible that the oligomerization principle discovered here with DEC-205 could provide insight into a family wide trait of an monomer–oligomer transition. Our data combined with preexisting studies thus suggest the role of oligomerization state impacts ligand binding of MR family proteins *in vitro*. We speculate that the oligomeric status of these endocytic receptors is either prerequisite for ligand binding or ligand dissociation within the endosomes. Considering DEC-205 is structurally pH-labile and that both CpG and keratin binding is pH-dependent (14), we hypothesize that cell-surface tetrameric DEC-205 could be a “cache” that monomerizes upon environmental cues, where pH is one to initiate antigen internalization. As DEC-205 is thought to be a sensor of apoptosis, necrosis, and bacterial infection, it could be that local acidification or aberrant pH triggers DEC-205 monomerization, thus enabling ligand capture before endocytosis. More research will be needed to elucidate the functional consequences of such receptor allostery and the impact on cellular activation once more DEC-205 ligands are identified and the signals that initiate internalization become clearer.

In summary, we have solved the high-resolution structure of a member of the macrophage MR family. The DEC-205 structure shows the concatenated arrangement of long CTLD stretches—previously thought impossible for structural analysis—with the N-terminal CysR and FNII domains sat atop the central link. Further, we solved the structure of the DEC-205 tetramer and showed major remodeling of the second CTLD ring under alkaline conditions. We also showed the presence of oligomeric DEC-205 form upon the cell surface at neutral pH and provided initial insights into the impact of oligomerization on CpG binding. Our data suggest a broad-reaching observation for ligand binding by members of the MR family with implications for endocytosis.

## Experimental procedures

### Soluble DEC-205 protein expression and purification

The cDNA encoding the human DEC-205 ECD (CD205; NP_002340, residues 1–1664) in-frame with a C-terminal FLAG tag (sequence DYKDDDK) and biotinylation consensus sequence (NSGLHHILDAQKMVWNHR) was synthesized by GeneArt/ThermoFisher. The DNA was codon-optimized for expression in human cells and cloned into the pcDNA3.1+ vector (ThermoFisher). Secreted protein was expressed by polyethylenime transient transfection of human embryonic kidney (HEK) 293S cells totaling four 1900 cm^2^ roller bottles of confluent cells each transfected with 600 μg of DNA. Following tangential flow filtration into TBS (10 mM Tris-HCl pH 8.0, 150 mM NaCl), the protein was loaded onto a FLAG-resin column overnight at 4 °C. Following column washing, DEC-205 was eluted with FLAG peptide and peak fractions collated and concentrated before a final clean-up over SE in either TBS, HBS (10 mM HEPES pH at 7.4 and 150 mM NaCl), or BIS-TRIS buffer (10 mM BIS-TRIS at pH 6.0 and 150 mM NaCl).

### Size exclusion–coupled multiangle light scattering

50 μl of DEC-205 ECD at 1 mg ml^−1^ was loaded onto a Superdex 200 5/150 column (GE Healthcare) in either TBS, HBS, or BIS-TRIS buffer at a flow rate of 0.3 ml min^−1^. The system comprises of a DGU-20A5 degasser, LC-20AD liquid chromatography, SIL-20ACHT autosampler, CBM-20A communications bus module, SPD-20A UV-visual detector, and CTO-20AC column oven (Shimadzu) coupled with a DAWN HELIOS-II light scattering detector and Optilab T-rEX refractive index detector (Wyatt). Detector number 12 was substituted for a WyattQELS detector installed at a 90° angle. The system was controlled using LC solutions (Shimadzu), and data collection and analysis were performed in ASTRA6 (Wyatt Technology Corp).

### Analytical ultracentrifugation

Oligomerization state and molecular weight assessments of DEC-205 ECD in solution were assessed via sedimentation velocity experiments using an Optima analytical ultracentrifuge (Beckman Coulter). DEC-205 at 0.2 mg ml^−1^ in either TBS buffer, HBS, or BIS-TRIS buffer was loaded into a dual-compartment cell next to 400 μl reference solution. All experiments were performed using an 8-hole rotor at 37,000 RPM at 20 °C, and the sedimentation velocity profiles were collected at wavelengths ranging from 230 to 310 nm. The collected data were analyzed in SEDFIT with a c(*s*) distribution model with a maximum entropy regularization of *p* = 0.68. The buffer density (1.0047 g ml^−1^) and viscosity (0.01002 cP) as well as sample frictional ratio (*f/f0*) were calculated from the SEDNTERP program using the primary amino acid sequence.

### Cryo-EM sample preparation and data collection parameters

Cryo-EM grids of purified DEC-205 ECD monomers were prepared by applying 3 μl of DEC-205 in BIS-TRIS buffer to copper 200 mesh Quantifoil R1.2/1.3 holey-carbon grids (ProSciTech) at a concentration of 0.3 mg ml^−1^. Using a Vitribot Mark IV, sample was applied to the grids at 4 °C and after ∼20s were blotted for 2s at −2 blot force before immediate vitrification in liquid ethane. Grids for the DEC-205 tetramer were prepared by applying 3 μl of DEC-205 ECD in TBS buffer at a concentration of 0.1 mg ml^−1^ and blotted as described above.

### Cryo-EM data collection parameters

Frozen grids were transferred to a Titan Krios transmission electron microscope (FEI) operated at 300 kV, equipped with a Quantum energy filter (Gatan) and Summit K2 (Gatan) detector. Automatic data acquisition was performed using EPU (FEI). Briefly, for the DEC-205 monomer, 12 s exposures through a defocus range of −0.8 to −2.8 μM were dose fractioned into 25 frame movies collected in energy filtered mode using a slit width of 20 eV at a nominal magnification of 130,000 × (energy filtered transmission electron microscopy), corresponding to a super resolution pixel size of 0.53 Å with a total dose of 53 electrons per Å^2^. Similarly for the DEC-205 tetramer, 7.2 s exposures through a defocus range of −0.8 to −3.5 μM were dose fractioned into 18-frame movies collected at 130,000 × magnification, corresponding to a pixel size of 1.06 Å with a total dose of 63 electrons per Å^2^.

### Image processing and map generation

Following data collection, dose-fractionated movies were aligned, corrected for beam-induced motion, dose weighted, averaged, and Fourier-binned × 2 within MotionCor2 (35). Estimation of the contrast transfer function parameters was made using Gctf (36) software. Automated particle picking was conducted using the Gautomatch software from Kai Zhang to determine the particle coordinates. The resultant particles were subjected to reference-free 2D classification using RELION 2.0 (37). Iterative rounds of particle classification yielded high-resolution 2D classes. In parallel, we undertook processing using the cryoSPARC processing pipeline (38), and using this approach, a suitable reference-free *ab initio* initial model was generated. The well-defined particles from clean 2D classifications were used for Initial 3D classification followed by initial 3D refinement. Following particle polishing, 3D classification with an increased Tau factor, T=(4), further separated the higher-resolution particles leading to a final refined and sharpened reconstruction at 3.2 Å. For the DEC-205 tetramer, the processing proceeded *via* the same workflow until 2D classification, and here, the high-resolution classes displayed an obvious D2 symmetry. The symmetry axes were aligned using Relion 3.0 (39) followed by subsequent 3D classifications with D2 symmetry imposed. The well-defined particles from clean 3D classifications were used for initial 3D refinement, particle polishing, and final 3D classification with increased Tau to further identify the higher-resolution tetrameric particles resulting in a final refined and sharpened reconstruction at 4.9 Å.

### Atomic model building and refinement

Upon finalization of the respective cryo-EM maps, crystal structures of the previously solved Cys-R (PDB 1DQO) (19), FN (PDB 2V5P) (40), and CTLD (PDB 1QDD) (41) were used as starting models for domain placement using Molrep from the CCP4-EM package (42, 43). Using *Coot* (44), the domains and linkers were built iteratively for the DEC-205 monomer before real-space refinement in Phenix including calculation of model to map correlation statistics (45, 46). Regions and sidechains with any ambiguity were removed, and the final model was validated using the Molprobity (47) and PDB validation service server (https://validate-rcsb-1.wwpdb.org/). The final DEC-205-monomer was then utilized for domain placements for the DEC-205 tetramer followed by rigid-body real-space refinement in Phenix.

### Confocal microscopy DEC-205–CpG co-localization studies

For confocal microscopy experiments, HEK293T cells were plated out onto a 35 mm FluoroDish (WPI Inc, Sarasota, FL) and cultured in DMEM supplemented with 10% (v/v) fetal bovine serum (FBS). Cells were transfected using lipofectamine 3000 and 14 μg of DNA of either full-length DEC-205 (CD205; NP_002340, residues 1–1722) or CD80 (NP_005182.1, residues 1–288) cloned into pcDNA-3.1+ in frame with a C-terminal eGFP. Before imaging, the media was switched to OPTI-MEM–phenol-free media supplemented with 2% (v/v) FBS. For the CpG co-localization study, DEC205_gfp_^+^ HEK293T cells were stained with Hoechst 33342 (1 μg ml^−1^) DNA marker, then incubated with 5 μM of either CpG or a nonthioated CpG control for 5 min before imaging. The following phosphorothioated 3′ labeled Atto647N 1668 CpG oligonucleotides were purchased from Geneworks: 5′TCC ATG ACG TTC CTG ATG CT as well as a nonthioated control. Images were acquired using a Zeiss LSM 980 Airyscan 2 (Zeiss, Jena, Germany) with a C-PlanApo 63x/1.4NA oil objective and an environmental (37 °C with 5% CO_2_ supplied) chamber.

### Number and brightness confocal analysis

For N&B experiments, HEK293T cells were plated out onto a 35 mm FluoroDish (WPI Inc, Sarasota, FL) cultured in DMEM supplemented with 10% (v/v) FBS and transfected as above. Before imaging, the media was switched to OPTI-MEM–phenol-free media supplemented with 2% (v/v) FBS. Confocal images were collected 16 h after transfection using a Fluoview1000 CLSM (Olympus, Tokyo, Japan) equipped with a 488 nm laser and 60× NA 1.2 water immersion lens in combination with an environmental (37 °C and 5% CO_2_ supplied) chamber (Olympus) equipped with a 488 nm laser and 60×/1.2NA water immersion lens. Images for N&B analysis were captured with the 488 nm laser attenuated to 0.1% laser power, PMT detector set to photon-counting mode, scan speed 12.5 *μ*s/pixel, scan area 256 × 256 pixels (50 nM pixel size), and 100 frames were captured per image series (27).

N&B analysis was performed using the SimFCS software (Laboratory for Fluorescence Dynamics, Irvine, CA) to determine the number (*N*) of diffusing particles within the focal spot and the intrinsic brightness (*B*) of each particle (27). Soluble eGFP was prepared and used as a brightness standard to determine the brightness (*B*) of monomeric eGFP (B_monomer_ = 1.3), being imaged under the same conditions as DEC-205–EGFP and CD80-eGFP; S_factor_ for the experimental setup was determined to be 1.3. The brightness of the eGFP was used to indicate the monomers (1.3 ± 0.15), dimers (1.6 ± 0.15), and tetramers (2.2 ± 0.15) on the Bmap and for further comparative analysis of oligomeric states (27).

### ELISA

ELISA plates were coated overnight at 4 °C with 10 μg ml^−1^ of MMRI7 anti-DEC-205 mAb (BD Biosciences). Unbound mAb has washed away (PBS, 0.05% [v/v] Tween-20) before proteins (diluted in PBS 3% [w/v] bovine serum albumin) were added (4 °C, overnight). Captured protein was exposed to various biotinylated CpG oligonucleotide at specified concentrations (2 h, room temperature), detected with streptavidin-horseradish peroxidase (2 h, room temperature) (GE Healthcare) and colorimetric reaction visualized using ABTS (2,2′-azino-bis-(3-ethylbenzothiazoline-6-sulphonic acid). Each titration point was performed in duplicate.

## Data and materials availability

PDB 7JPT and 7JPU and EMDB codes 22422 and 22423 for the monomer and tetramer, respectively.

## Conflicts of interest

The authors declare that they have no conflicts of interest with the contents of this article.

## References

[1] Wileman T.E., Lennartz M.R., Stahl P.D. (1986). Identification of the macrophage mannose receptor as a 175-kDa membrane protein. Proc. Natl. Acad. Sci. U. S. A..

[2] Lambeau G., Ancian P., Barhanin J., Lazdunski M. (1994). Cloning and expression of a membrane receptor for secretory phospholipases A2. J. Biol. Chem..

[3] Wu K., Yuan J., Lasky L.A. (1996). Characterization of a novel member of the macrophage mannose receptor type C lectin family. J. Biol. Chem..

[4] Jiang W., Swiggard W.J., Heufler C., Peng M., Mirza A., Steinman R.M., Nussenzweig M.C. (1995). The receptor DEC-205 expressed by dendritic cells and thymic epithelial cells is involved in antigen processing. Nature.

[5] East L., Isacke C.M. (2002). The mannose receptor family. Biochim. Biophys. Acta.

[6] Inaba K., Swiggard W.J., Inaba M., Meltzer J., Mirza A., Sasagawa T., Nussenzweig M.C., Steinman R.M. (1995). Tissue distribution of the DEC-205 protein that is detected by the monoclonal antibody NLDC-145. I. Expression on dendritic cells and other subsets of mouse leukocytes. Cell Immunol..

[7] Carbone F.R., Belz G.T., Heath W.R. (2004). Transfer of antigen between migrating and lymph node-resident DCs in peripheral T-cell tolerance and immunity. Trends Immunol..

[8] Mahnke K., Guo M., Lee S., Sepulveda H., Swain S.L., Nussenzweig M., Steinman R.M. (2000). The dendritic cell receptor for endocytosis, DEC-205, can recycle and enhance antigen presentation via major histocompatibility complex class II–positive lysosomal compartments. J. Cell Biol..

[9] Taylor M.E. (1997). Evolution of a family of receptors containing multiple C-type carbohydrate-recognition domains. Glycobiology.

[10] Lahoud M.H., Ahmet F., Zhang J.G., Meuter S., Policheni A.N., Kitsoulis S., Lee C.N., O'Keeffe M., Sullivan L.C., Brooks A.G., Berry R., Rossjohn J., Mintern J.D., Vega-Ramos J., Villadangos J.A. (2012). DEC-205 is a cell surface receptor for CpG oligonucleotides. Proc. Natl. Acad. Sci. U. S. A..

[11] Davila E., Celis E. (2000). Repeated administration of cytosine-phosphorothiolated guanine-containing oligonucleotides together with peptide/protein immunization results in enhanced CTL responses with anti-tumor activity. J. Immunol..

[12] Shrimpton R.E., Butler M., Morel A.-S., Eren E., Hue S.S., Ritter M.A. (2009). CD205 (DEC-205): a recognition receptor for apoptotic and necrotic self. Mol. Immunol..

[13] Cao L., Shi X., Chang H., Zhang Q., He Y. (2015). pH-Dependent recognition of apoptotic and necrotic cells by the human dendritic cell receptor DEC205. Proc. Natl. Acad. Sci. U. S. A..

[14] Cao L., Chang H., Shi X., Peng C., He Y. (2016). Keratin mediates the recognition of apoptotic and necrotic cells through dendritic cell receptor DEC205/CD205. Proc. Natl. Acad. Sci. U. S. A..

[15] He Y., Bjorkman P.J. (2011). Structure of FcRY, an avian immunoglobulin receptor related to mammalian mannose receptors, and its complex with IgY. Proc. Natl. Acad. Sci. U. S. A..

[16] Dong Y., Cao L., Tang H., Shi X., He Y. (2017). Structure of human M-type phospholipase A2 receptor revealed by cryo-electron microscopy. J. Mol. Biol..

[17] Boskovic J., Arnold J.N., Stilion R., Gordon S., Sim R.B., Rivera-Calzada A., Wienke D., Isacke C.M., Martinez-Pomares L., Llorca O. (2006). Structural model for the mannose receptor family uncovered by electron microscopy of Endo180 and the mannose receptor. J. Biol. Chem..

[18] Feinberg H., Park-Snyder S., Kolatkar A.R., Heise C.T., Taylor M.E., Weis W.I. (2000). Structure of a C-type carbohydrate recognition domain from the macrophage mannose receptor. J. Biol. Chem..

[19] Liu Y., Chirino A.J., Misulovin Z., Leteux C., Feizi T., Nussenzweig M.C., Bjorkman P.J. (2000). Crystal structure of the cysteine-rich domain of mannose receptor complexed with a sulfated carbohydrate ligand. J. Exp. Med..

[20] Hu Z., Shi X., Yu B., Li N., Huang Y., He Y. (2018). Structural insights into the pH-dependent conformational change and collagen recognition of the human mannose receptor. Structure.

[21] Fiete D.J., Beranek M.C., Baenziger J.U. (1998). A cysteine-rich domain of the “mannose” receptor mediates GalNAc-4-SO4 binding. Proc. Natl. Acad. Sci. U. S. A..

[22] Paracuellos P., Briggs David C., Carafoli F., Lončar T., Hohenester E. (2015). Insights into collagen uptake by C-type mannose receptors from the crystal structure of Endo180 domains 1-4. Structure.

[23] Zelensky A.N., Gready J.E. (2005). The C-type lectin-like domain superfamily. FEBS J..

[24] Ryan J.C., Turck J., Niemi E.C., Yokoyama W.M., Seaman W.E. (1992). Molecular cloning of the NK1.1 antigen, a member of the NKR-P1 family of natural killer cell activation molecules. J. Immunol..

[25] Iizuka K., Naidenko O.V., Plougastel B.F.M., Fremont D.H., Yokoyama W.M. (2003). Genetically linked C-type lectin-related ligands for the NKRP1 family of natural killer cell receptors. Nat. Immunol..

[26] Adámková L., Kvíčalová Z., Rozbeský D., Kukačka Z., Adámek D., Cebecauer M., Novák P. (2019). Oligomeric architecture of mouse activating Nkrp1 receptors on living cells. Int. J. Mol. Sci..

[27] Digman M.A., Dalal R., Horwitz A.F., Gratton E. (2008). Mapping the number of molecules and brightness in the laser scanning microscope. Biophys. J..

[28] Wang R., Qi X., Schmiege P., Coutavas E., Li X. (2020). Marked structural rearrangement of mannose 6-phosphate/IGF2 receptor at different pH environments. Sci. Adv..

[29] Hijazi K., Wang Y., Scala C., Jeffs S., Longstaff C., Stieh D., Haggarty B., Vanham G., Schols D., Balzarini J., Jones I.M., Hoxie J., Shattock R., Kelly C.G. (2011). DC-SIGN increases the affinity of HIV-1 envelope glycoprotein interaction with CD4. PLoS One.

[30] Snyder G.A., Ford J., Torabi-Parizi P., Arthos J.A., Schuck P., Colonna M., Sun P.D. (2005). Characterization of DC-SIGN/R interaction with human immunodeficiency virus type 1 gp120 and ICAM molecules favors the receptor's role as an antigen-capturing rather than an adhesion receptor. J. Virol..

[31] Taylor M.E., Drickamer K. (1993). Structural requirements for high affinity binding of complex ligands by the macrophage mannose receptor. J. Biol. Chem..

[32] Napper C.E., Dyson M.H., Taylor M.E. (2001). An extended conformation of the macrophage mannose receptor. J. Biol. Chem..

[33] Roseman D.S., Baenziger J.U. (2000). Molecular basis of lutropin recognition by the mannose/GalNAc-4-SO4 receptor. Proc. Natl. Acad. Sci. U. S. A..

[34] Lai J., Bernhard O.K., Turville S.G., Harman A.N., Wilkinson J., Cunningham A.L. (2009). Oligomerization of the macrophage mannose receptor enhances gp120-mediated binding of HIV-1. J. Biol. Chem..

[35] Li X., Mooney P., Zheng S., Booth C.R., Braunfeld M.B., Gubbens S., Agard D.A., Cheng Y. (2013). Electron counting and beam-induced motion correction enable near-atomic-resolution single-particle cryo-EM. Nat. Methods.

[36] Zhang K. (2016). Gctf: real-time CTF determination and correction. J. Struct. Biol..

[37] Kimanius D., Forsberg B.O., Scheres S.H.W., Lindahl E. (2016). Accelerated cryo-EM structure determination with parallelisation using GPUs in RELION-2. eLife.

[38] Punjani A., Rubinstein J.L., Fleet D.J., Brubaker M.A. (2017). cryoSPARC: algorithms for rapid unsupervised cryo-EM structure determination. Nat. Methods.

[39] Zivanov J., Nakane T., Forsberg B.O., Kimanius D., Hagen W.J.H., Lindahl E., Scheres S.H.W. (2018). New tools for automated high-resolution cryo-EM structure determination in RELION-3. eLife.

[40] Brown J., Delaine C., Zaccheo O.J., Siebold C., Gilbert R.J., van Boxel G., Denley A., Wallace J.C., Hassan A.B., Forbes B.E., Jones E.Y. (2008). Structure and functional analysis of the IGF-II/IGF2R interaction. EMBO J..

[41] Gerbaud V., Pignol D., Loret E., Bertrand J.A., Berland Y., Fontecilla-Camps J.-C., Canselier J.-P., Gabas N., Verdier J.-M. (2000). Mechanism of calcite crystal growth inhibition by the N-terminal undecapeptide of lithostathine. J. Biol. Chem..

[42] Wood C., Burnley T., Patwardhan A., Scheres S., Topf M., Roseman A., Winn M. (2015). Collaborative computational project for electron cryo-microscopy. Acta Crystallogr. D.

[43] Burnley T., Palmer C.M., Winn M. (2017). Recent developments in the CCP-EM software suite. Acta Crystallogr. D.

[44] Emsley P., Lohkamp B., Scott W.G., Cowtan K. (2010). Features and development of Coot. Acta Crystallogr. D.

[45] Afonine P.V., Poon B.K., Read R.J., Sobolev O.V., Terwilliger T.C., Urzhumtsev A., Adams P.D. (2018). Real-space refinement in PHENIX for cryo-EM and crystallography. Acta Crystallogr. D.

[46] Liebschner D., Afonine P.V., Baker M.L., Bunkoczi G., Chen V.B., Croll T.I., Hintze B., Hung L.-W., Jain S., McCoy A.J., Moriarty N.W., Oeffner R.D., Poon B.K., Prisant M.G., Read R.J. (2019). Macromolecular structure determination using X-rays, neutrons and electrons: recent developments in Phenix. Acta Crystallogr. D.

[47] Williams C.J., Headd J.J., Moriarty N.W., Prisant M.G., Videau L.L., Deis L.N., Verma V., Keedy D.A., Hintze B.J., Chen V.B., Jain S., Lewis S.M., Arendall W.B., Snoeyink J., Adams P.D. (2018). MolProbity: more and better reference data for improved all-atom structure validation. Protein Sci..

