# Supplementary material for: The cryo-EM structure of the endocytic receptor DEC-205
Source: J Biol Chem. 2020 Dec 3;296:100127. doi: 10.1074/jbc.RA120.016451 (PMC7948739; doi:10.1074/jbc.RA120.016451)
Supplement: Supplementary Figures and Tables [file mmc1.pdf]

**Supplementary Materials**

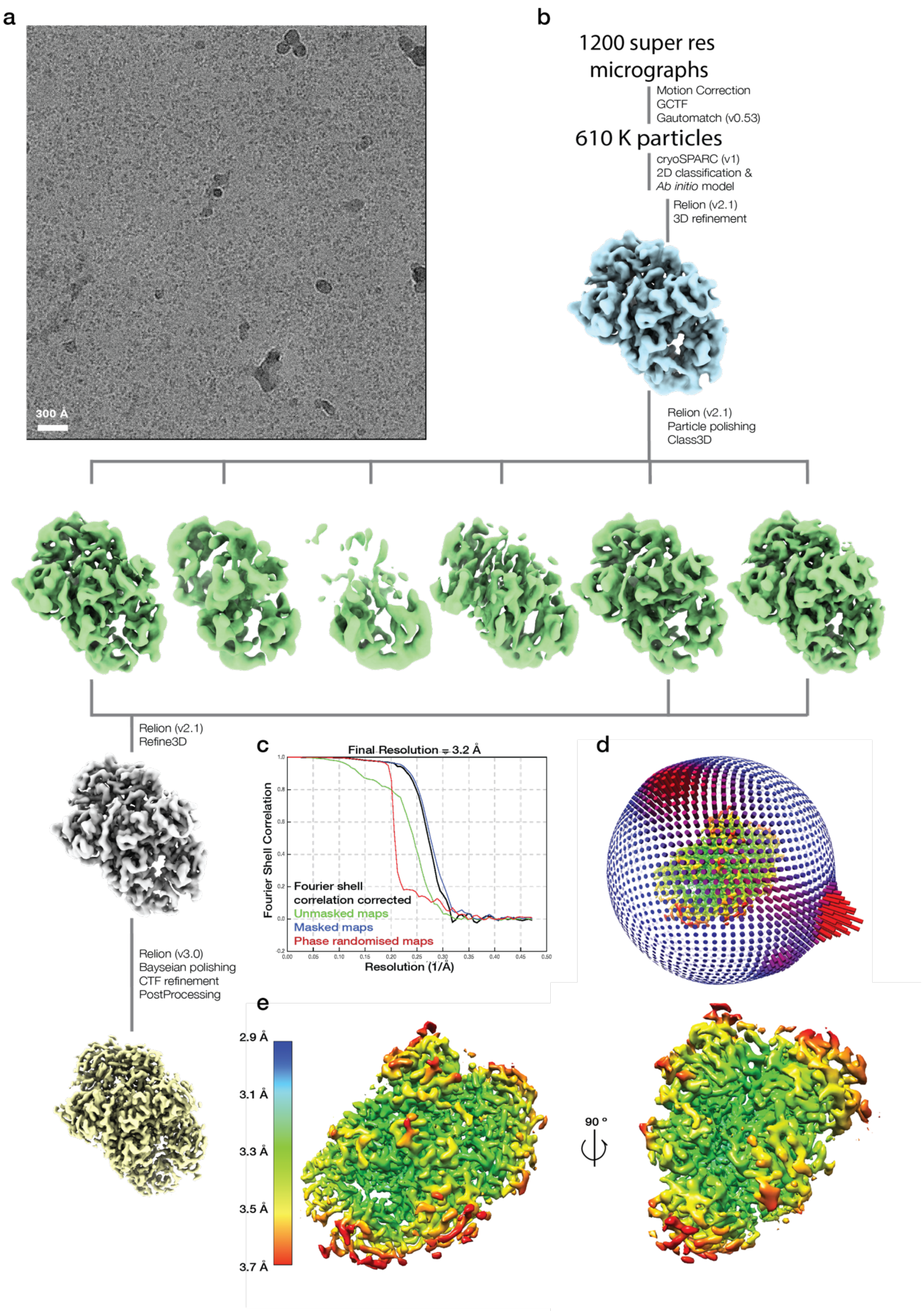


**Figure S1. Cryo-EM data processing and workflow of the DEC-205 monomer. (a)** A

representative micrograph of the DEC-205 monomer. (**b**) Cryo-EM data processing workflow. (**c**) Gold-standard Fourier shell correlation (FSC) curves showing a nominal global resolution of 3.2 Å. (**d**) 3D histogram representation of the Euler angle distribution the particles used in the final reconstruction shown overlaid onto the reconstructed map coloured according to local resolution. (**e**) Final postprocessed map coloured according to local resolution.

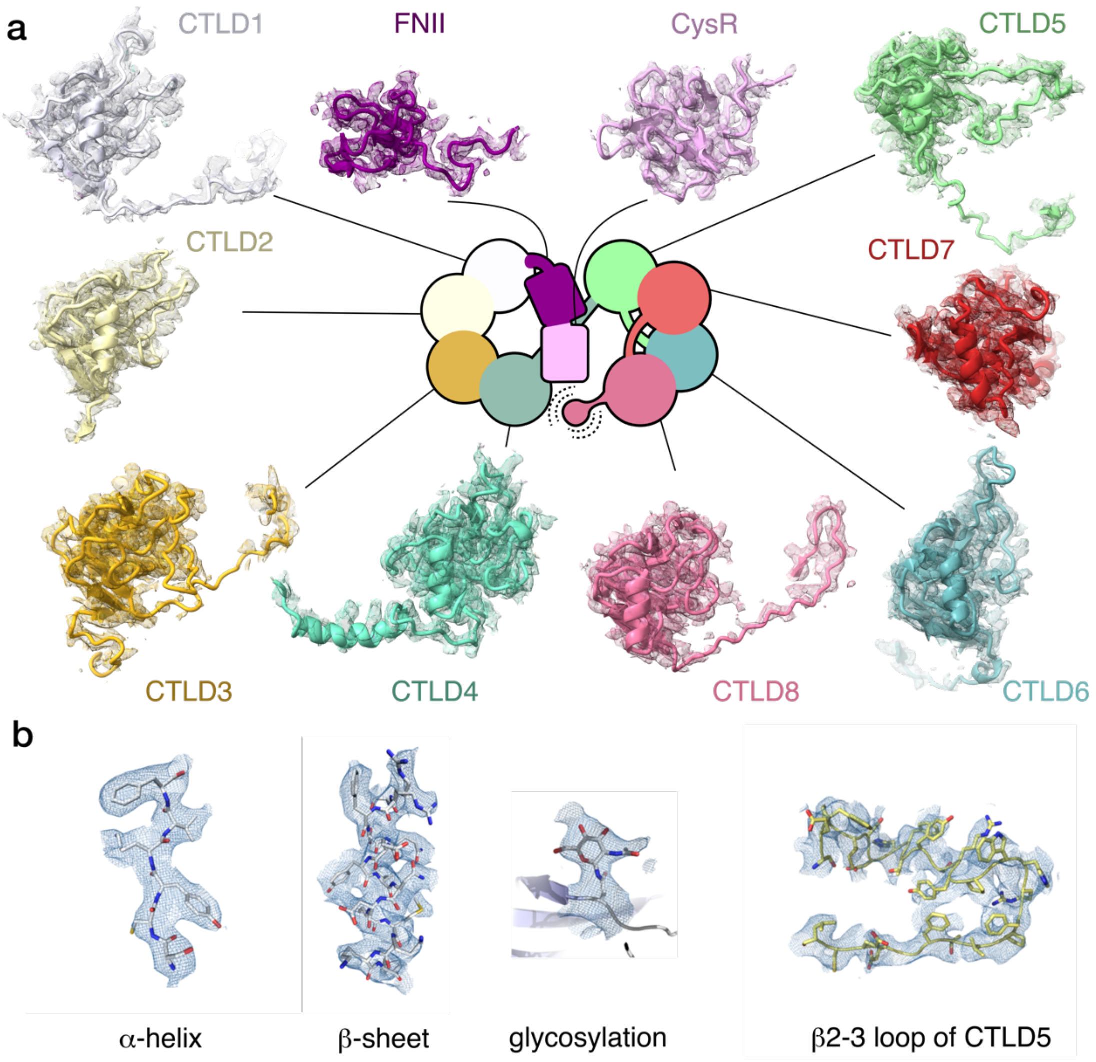


**Figure S2. Cryo-EM domain density throughout the DEC-205 reconstruction. (a)** Cryo-EM map and models for the DEC-205 domains. **(b)** Example density of an α-helix, β-sheet, glycosylation and the extended β2-3 loop of CTLD5.

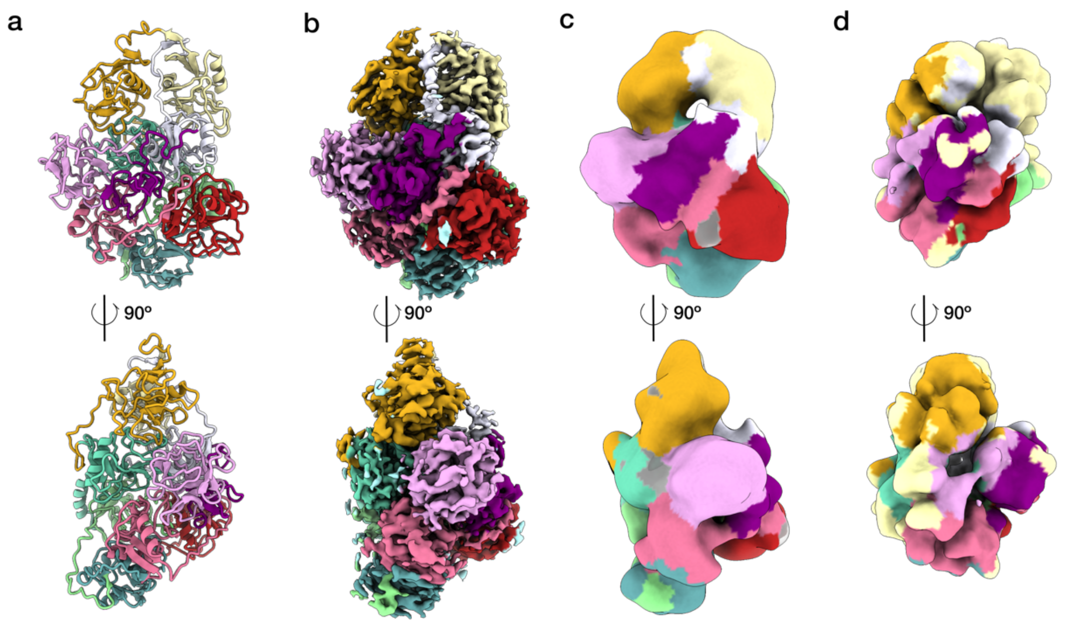


**Figure S3. Comparison of the DEC-205 cryo-EM reconstruction to prior map.** (**a**) Cartoon representation of the monomer DEC-205 and the comparative view of the reconstructed map (**b**), domains coloured Lavender (CysR), Purple (FN-II), Light blue (CTLD1), pale yellow (CTLD2), gold (CTLD3), mint (CTLD4), light green (CTLD5), light blue (CTLD6), pink (CTLD7), and red (CTLD8). We generated a gaussian contoured map of the 3.2 Å DEC-205 reconstruction (**c**), to match the resolution of the reconstruction published previously by Cao et al. PNAS 2015, (**d**).

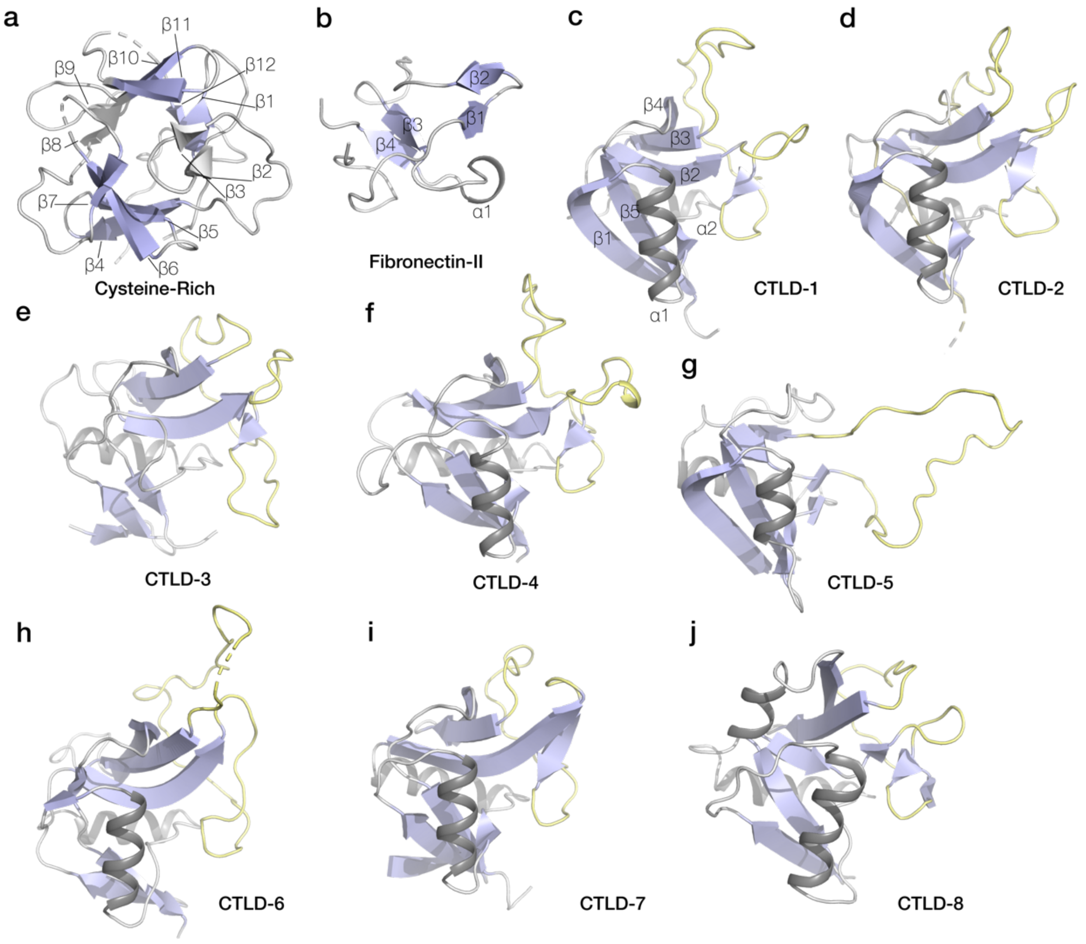


**Figure S4. Expanded analysis of the DEC-205 domains.** The extracellular domains of the DEC-205 monomer shown as cartoon including the Cysteine-Rich, (**a**) and Fibronectin domains, (**b**) with the α-helices (dark grey), β-sheets (light blue) and the extended or compact forms of the β2-3 loops (yellow) for CTLD1 (**c**), CTLD2 (**d**), CTLD3 (**e**), CTLD4 (**f**), CTLD5 (**g**), CTLD6 (**h**), CTLD7 (**i**) and CTLD8 (**j**).

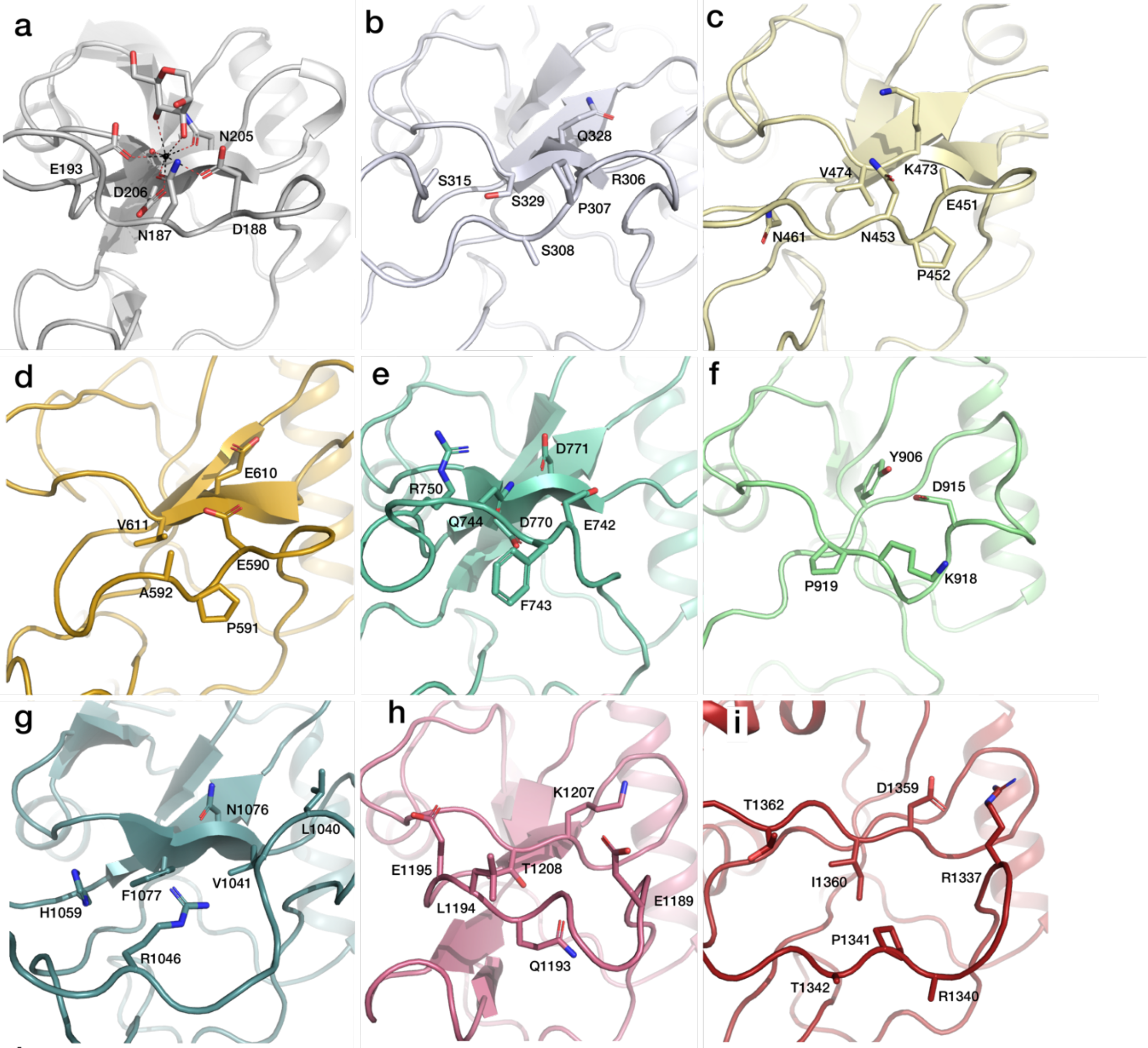


j

```
ratMBP-A   179    SNWKKD--EPNDHGS--------GEDCVTI-VD--N---GLWNDISCQ   238
CTLD1      300    LNWDPD--RPSAPTI-------GGSSCARMDAE--S---GLWQSFSCE   333
CTLD2      445    TYWDEN--EPNVPYN-------KTPNCVSYLGE--L---GQWKVQSCE   478
CTLD3      548    SNWNFL--EPASPG-----------GCVAMST---GKSVGKWEVKDCR   615
CTLD4      735    STIIMPN-EFQQD--------YDIRDCAAVKVF-HRPWRRGWHFYDDR   772
CTLD5      890    RYP-----HRFPVT--------FGEECLYMSAK--TWLIDLGKPTDCS   923
CTLD6     1034    SNFHPLLVSGRLRIPENFFEEESRYHCALILNLQKSPFTGTWNFTSCS  1081
CTLD7     1185    SRWA----ETNG----------QLEDCVV------LDTDGFWKTVDCN  1212
CTLD8     1332    SYTHWRAGRPTIKN--------EKFLAG-------LSTDGFWDIQTFK  1364
```

**Figure S5. Analysis of the DEC-205 CTLD β2-3 loops.** (**a**) Comparison and alignments of the β2-3 loop of the rat MBP CTLD with coordinated calcium imparting sugar binding capacity. Comparative perspectives of the DEC-205 CTLD1 (**b**), CTLD2 (**c**), CTLD3 (**d**), CTLD4 (**e**), CTLD5 (**f**), CTLD6 (**g**), CTLD7 (**h**) and CTLD8 (**i**) shows low structural conservation at this site corroborating the low sequence amino acid conservation (**j**).

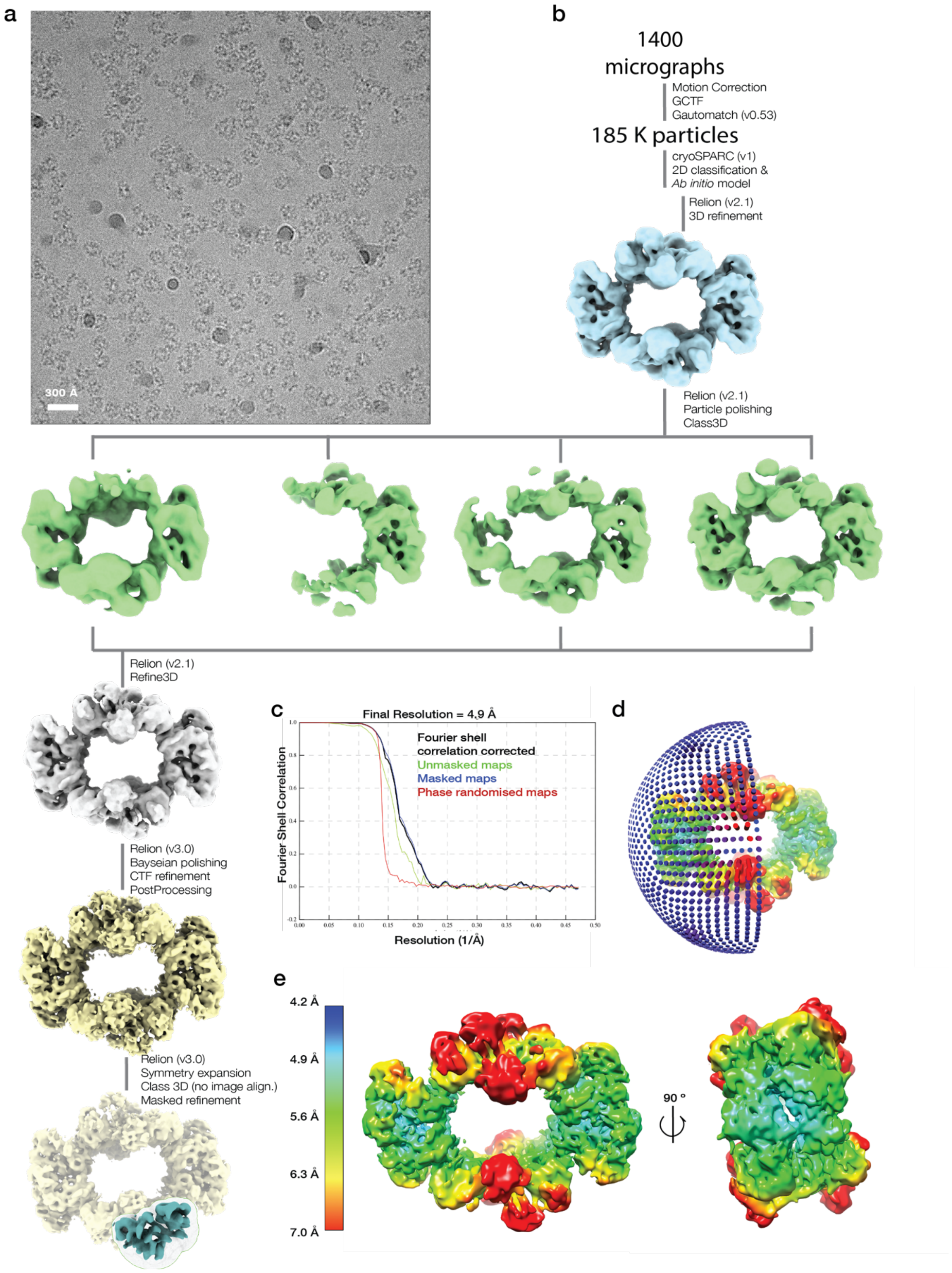


**Figure S6. Cryo-EM data processing and workflow of the DEC-205 tetramer.** (**a**) A representative micrograph of the DEC-205 tetramer. (**b**) Cryo-EM data processing workflow. (**c**) Gold-standard Fourier shell correlation (FSC) curves showing a nominal global resolution of 4.9Å. (**d**) 3D histogram representation of the Euler angle distribution the particles used in the final reconstruction shown overlaid onto the reconstructed map coloured according to local resolution. (**e**) Final postprocessed map coloured according to local resolution.

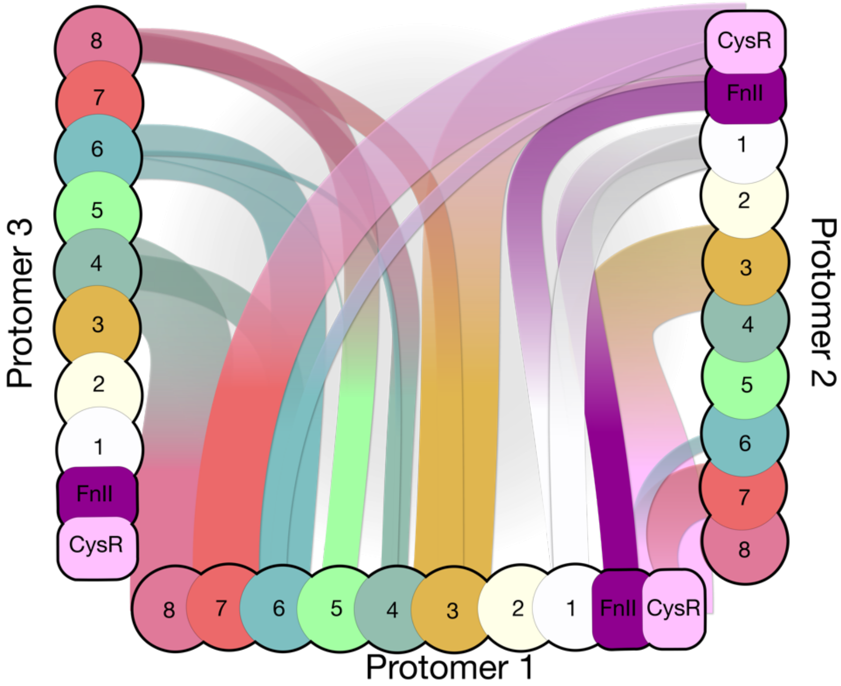


**Figure S7. Buried surface area analysis of the DEC-205 tetramer.** Representation of the buried surface interactions of the DEC-205 protomer 1 with protomers 2 & 3 within the tetramer. The width of the line corresponds to the BSA in $Å^2$ as derived from the PDBePISA software.

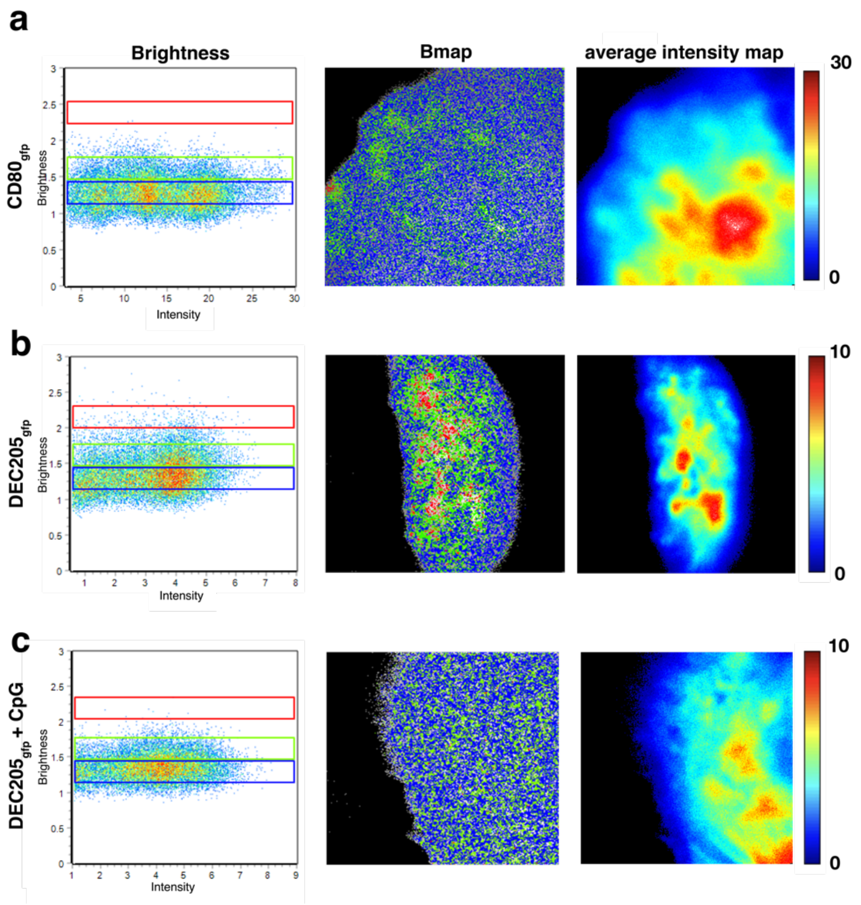


**Figure S8. Cell surface Number and Brightness microscopy.** Representative Number and Brightness (N&B) microscopy analysis of (**a**) $CD80_{gfp}^{+}$ , (**b**) DEC-$205_{gfp}^{+}$ and DEC-$205_{gfp}^{+}$ + CpG (**c**) HEK293T cells. Brightness vs Intensity plots (left) highlights monomers (blue), dimers (green) and tetramers (red) visualised in the Bmap (centre) followed by the average intensity map (right). Although both $CD80_{gfp}^{+}$ and DEC-$205_{gfp}^{+}$ cells exhibited similar average intensity patterns, DEC-205 clearly exists in larger oligomeric assemblies on a cell surface, shown green and red, in either a diffuse pattern or in punctate clusters. Scan area 256 × 256 pixels (50 nM pixel size).

## Table S1 | Inter-domain buried surface areas within the monomer (Å$^2$)

Table S1 | Inter-domain buried surface areas within the monomer (Å$^2$)

| | CysR | FNII | CTLD1 | CTLD2 | CTLD3 | CTLD4 | CTLD5 | CTLD6 | CTLD7 | CTLD8 |
|---|---|---|---|---|---|---|---|---|---|---|
| **CysR** | | 462.9 | 55.5 | | 102.4 | 432.9 | | | 440.5 | |
| **FNII** | 513.6 | | 179.2 | | | 48.6 | 75.8 | | 120.5 | 183.2 |
| **CTLD1** | 57.6 | 154.4 | | 880.2 | 35.1 | 979.3 | 353.8 | | | 564.5 |
| **CTLD2** | | | 832.3 | | 197.8 | 73.6 | | | | |
| **CTLD3** | | 105.4 | 36.8 | 219.5 | | 607.2 | | | | |
| **CTLD4** | | 56.7 | 1014.9 | 76.6 | 667.9 | | 233.2 | | 421.5 | |
| **CTLD5** | | 71.1 | 343.6 | | | 222.2 | | 1134.8 | 345 | 527.1 |
| **CTLD6** | | | | | | | 1150.9 | | 637.6 | 286.6 |
| **CTLD7** | 431.1 | 161.2 | | | | 482.7 | 307.9 | 670.1 | | |
| **CTLD8** | | 140.2 | 607.8 | | | | 538.4 | 278.4 | | |

## Table S2 | Inter-domain buried surface areas within the tetramer (Å$^2$)

Table S2 | Inter-domain buried surface areas within the tetramer (Å$^2$)

| | | Protomer A | | | | | | | | | |
|---|---|---|---|---|---|---|---|---|---|---|---|
| | | **CysR** | **FNII** | **CTLD1** | **CTLD2** | **CTLD3** | **CTLD4** | **CTLD5** | **CTLD6** | **CTLD7** | **CTLD8** |
| **Protomer B** | **CysR** | | | | | 448.2 | | | 90.9 | 383.1 | |
| | **FNII** | | | 185.5 | | | | | | | |
| | **CTLD1** | | 202.0 | 199.0 | | | | | | | |
| | **CTLD2** | | | | | | | | | | |
| | **CTLD3** | 483.9 | | | | | | | | | |
| | **CTLD4** | | | | | | | | | | |
| | **CTLD5** | | | | | | | | | | |
| | **CTLD6** | 92.8 | | | | | | | | | |
| | **CTLD7** | 311.1 | | | | | | | | | |
| | **CTLD8** | | | | | | | | | | |
| **Protomer C** | **CysR** | | | | | | | | | | |
| | **FNII** | | | | | | | | | | |
| | **CTLD1** | | | | | | | | | | |
| | **CTLD2** | | | | | | | | | | |
| | **CTLD3** | | | | | | | | | | |
| | **CTLD4** | | | | | | | | 220.3 | | 472.7 |
| | **CTLD5** | | | | | | | | | | |
| | **CTLD6** | | | | | | 31.6 | 6.9 | | | |
| | **CTLD7** | | | | | | | | | | |
| | **CTLD8** | | | | | 143.0 | 152.7 | 199.5 | 315.5 | | |
